# Comprehensive pan-cancer investigation: unraveling the oncogenic, prognostic, and immunological significance of Abelson interactor family member 3 gene in human malignancies

**DOI:** 10.3389/fmolb.2023.1277830

**Published:** 2023-10-24

**Authors:** Aijun Sun, Fengze Cai, Qingping Xiong, Tong Xie, Xiang Li, Yanteng Xie, Ruiyang Luo, Wenwen Hu, Fei Zhong, Shiyan Wang

**Affiliations:** ^1^ Department of Thyroid and Breast Oncological Surgery, The Affiliated Huaian Hospital of Xuzhou Medical University and Huai’an Second People’s Hospital, Huai’an, Jiangsu, China; ^2^ School of Life Science and Food Engineering, Huaiyin Institute of Technology, Huai’an, Jiangsu, China; ^3^ Jiangsu Key Laboratory of Regional Resource Exploitation and Medicinal Research, Huaiyin Institute of Technology, Huai’an, Jiangsu, China; ^4^ Third Affiliated Hospital of Henan University of Traditional Chinese Medicine, Zhengzhou, Henan, China; ^5^ Department of Laboratory Medicine, The Affiliated Huaian Hospital of Xuzhou Medical University and Huai’an Second People’s Hospital, Huai’an, Jiangsu, China

**Keywords:** ABI3, pan-cancer, prognostic biomarker, immunotherapy, tumor immunity

## Abstract

**Background:** Abelson interactor Family Member 3 (ABI3) encodes protein that not only suppresses the ectopic metastasis of tumor cells but also hinders their migration. Although ABI3 had been found to modulate the advancement of diverse neoplasms, there is no comprehensive pan-cancer analysis of its effects.

**Methods:** The transcriptomics data of neoplasm and normal tissues were retrieved from the Genomic Data Commons (GDC) data portal, and UCSC XENA database. To gather protein information for ABI3, Human Protein Atlas (HPA) and GeneMANIA websites were utilized. Additionally, Tumor Immune Single-cell Hub (TISCH) database was consulted to determine the primary cell types expressing ABI3 in cancer microenvironments. Univariate Cox regression approach was leveraged to evaluate ABI3’s prognostic role across cancers. The Cbioportal and Gene Set Cancer Analysis (GSCA) website were leveraged to scrutinize the genomic landscape information across cancers. TIMER2.0 was leveraged to probe the immune cell infiltrations associated with ABI3 across cancers. The associations of ABI3 with immune-related genes were analyzed through Spearman correlation method. Gene Set Enrichment Analysis (GSEA) and Gene Set Variation Analysis (GSVA) were utilized to search associated biological pathways. The CellMiner database and molecular docking were implemented to identify potential interactions between the ABI3 protein and specific anticarcinogen.

**Findings:** ABI3 expression and its ability to predict prognosis varied distinct tumor, with particularly high expression observed in Tprolif cells and monocytes/macrophages. Copy number variation (CNV) and methylation negatively correlated with ABI3 expression in the majority of malignancies. Corresponding mutation survival analysis indicated that the mutation status of ABI3 was strongly connected to the prognosis of LGG patients. ABI3 expression was linked to immunotherapeutic biomarkers and response in cancers. ESTIMATE and immune infiltrations analyses presented ABI3 association with immunosuppression. ABI3 was significantly correlated with immunoregulators and immune-related pathways. Lastly, prospective ABI3-targeted drugs were filtered and docked to ABI3 protein.

**Interpretation:** Our study reveals that ABI3 acts as a robust tumor biomarker. Its functions are vital that could inhibit ectopic metastasis of tumor cells and modulate cellular adhesion and migration. The discoveries presented here may have noteworthy consequences for the creation of fresh anticancer suppressors, especially those targeting BRCA.

## 1 Introduction

The burgeoning prevalence and fatality figures of neoplastic diseases represent a substantial challenge to public wellbeing across the international landscape. Regrettably, to the present day, no definitive remedies for malignancies have been identified (Siegel et al., 2023). In spite of extensive endeavors to augment diagnostic and therapeutic capabilities, the survival outcomes for afflicted individuals continue to be dishearteningly inadequate (Ferlay et al., 2021). Consequently, an exigent demand exists for the discovery of diagnostic indicators and innovative therapeutic interventions for oncological conditions.

The emergence of cutting-edge sequencing methodologies coupled with bioinformatics progression has inaugurated novel opportunities to investigate the molecular panorama of neoplastic diseases. Sweeping cancer genomics endeavors, such as The Cancer Genome Atlas (TCGA) have amassed vast compendiums of oncological genomic and clinical information. These initiatives have expedited the recognition of tumorigenic impelling genes, molecular subclasses, and distinct characteristics, bestowing invaluable comprehension of cancer’s intricate biology (Blum et al., 2018).

By methodically amalgamating multi-omics data, pan-cancer gene expression examinations can be executed, permitting evaluations of correlations between gene expression profiles, clinical prognoses, and pertinent signaling cascades. This holistic methodology unveils substantial prospects for uncovering innovative immunotherapeutic targets and broadening our grasp of neoplastic biology. Moreover, this enriched understanding can inform diagnostic, prognostic, and therapeutic approaches, ultimately enhancing patient outcomes.

The Abelson interactor (ABI) protein family, encompassing Abi1, Abi2, and Abi3, plays a crucial role as a coordinator of Rac-mediated actin polymerization, which is essential for cellular adhesion and motility (Yu et al., 2008; Chen et al., 2014). In mammalian cells, Abi proteins form a complex with other proteins, such as WASP-family verprolin-homologous (WAVE), hematopoietic stem progenitor cell 300, Nck-associated protein, and Rac1-specific protein (Eden et al., 2002; Innocenti et al., 2004). This complex, known as the WAVE regulatory complex, modulates the actin nucleation factor actin-related protein 2/3 complex, thereby controlling actin dynamics at the forefront of migrating cells (Grove et al., 2004; Law et al., 2013; Schaks et al., 2019). By regulating cellular adhesion and migration, ABI family proteins have been extensively documented to have a significant impact on the progression of various malignancies, such as leukemia, colorectal cancer, and breast cancer (Wang et al., 2007; Chorzalska et al., 2014; Steinestel et al., 2014). Additionally, ABI family proteins may modulate melanoma through the Rac-WAVE2 signaling pathway (Kurisu et al., 2005). Among the ABI family members, ABI3 has been extensively studied and shown to significantly impact cancer progression.

The ABL-interactor 3 (ABI3) protein, also referred to as the novel SH3 domain-containing molecule, belonged to the ABI protein family (Leng et al., 2005). Numerous studies have substantiated the robustly correlation between ABI3 expression levels and cancer onset and progression (Matsuda et al., 2008; Kanduri et al., 2010; Pathania et al., 2015). Tumor dissemination was a multifaceted, dynamic process encompassing augmented migration towards remote locations (Tsai and Yang, 2013). The protein encoded by the ABI3 gene was implicated in impeding cancer cell dissemination and motility, potentially via interactions with essential intracellular entities, such as PAK, which play a role in cellular motility (Ichigotani et al., 2002). Notwithstanding its ubiquitous expression in healthy tissues, ABI3 expression was frequently reduced in invasive malignancies (Miyazaki et al., 2000). This downregulation was associated with various cancer types, encompassing thyroid tumors and colorectal carcinoma cell lines (Latini et al., 2011). Overall, ABI3 was among the most comprehensively investigated ABI family members in the context of cancer progression.

As the majority of investigations regarding ABI3’s involvement in neoplasms focusing on singular malignancies, a comprehensive pan-cancer examination of associations between ABI3 and diverse cancers remains unexplored. We employed an array of databases such as TCGA, HPA, CCLE, GTEx, and TISCH to scrutinize ABI3 expression levels and their prognostic implications across a spectrum of malignancies. Additionally, we conducted pan-cancer genomic alteration and prognostic assessments of ABI3, exploring potential links with CNV, DNA methylation, microsatellite instability (MSI), tumor mutational burden (TMB), and immune infiltration in neoplasms. The relationship between ABI3 immune checkpoint blockade therapy, using two independent immunotherapy cohorts, was also investigated. To elucidate the function of ABI3 in 33 types of tumors, we conducted co-expression analysis of ABI3 with stroma scores, immune scores, ESTIMATE scores, as well as mismatch repair (MMR) and immune-related genes. Moreover, we conducted GSEA, GSVA, and evaluations of drug sensitivity in various cancers.

Our findings substantiate ABI3 as a reliable prognostic indicator for numerous cancers, implicating its significant functions in tumor immunity through influencing immune cell infiltration, TMB, and MSI. Furthermore, our drug analysis findings reveal a correlation between ABI3 and the pharmacological agents employed in the management of BRCA, indicating that ABI3 may deem as a promising immunotherapy biomarker in this malignancy. This research paves the way for further exploration of ABI3’s role in cancer immunity.

## 2 Materials and methods

### 2.1 Data sources and processing methods

A bubble graph was initially presented to show the diseases or phenotypes associated with ABI3 from the Open Target Platform (https://platform.opentargets.org/). To affirm the subcellular distribution of ABI3 protein, the Human Protein Atlas (HPA: https://www.proteinatlas.org/) database was exploited. The RNA-seq data, represented in transcripts per million (TPM) format, were obtained from the UCSC XENA database (https://xenabrowser.net/datapages/?host=https%3A%2F%2Ftoil.xenahubs.net) for both TCGA samples and their corresponding normal tissue samples from GTEx. To ensure consistency, the data were subjected to uniform processing using the Toil pipeline (Vivian et al., 2017). ABI3 expression levels were assessed in 33 cancerous and their corresponding normal tissues using the downloaded data. To analyze the differences in mRNA expression of ABI3 between cancerous and adjacent normal tissues, the Gene_DE module of Tumor Immune Estimation Resource 2.0 (TIMER2.0) was leveraged (L et al., 2020). Data from each tumor cell line, obtained from the Cancer Cell Line Encyclopedia (CCLE) database (https://portals.broadinstitute.org/ccle/), and expression levels in 29 tissues were analyzed based on the cancer cells source (Nusinow et al., 2020). In addition to pan-cancer gene expression differential analysis, TPM-formatted mRNA expression data and relevant clinical data used for all subsequent analyses were derived from TCGA pan-cancer cohort datasets downloaded from the GDC data portal (Tomczak et al., 2015). To obtain ABI3 protein interaction information, GeneMANIA (http://www.genemania.org) was employed. This website constructs protein-protein interaction (PPI) networks that facilitate the prediction of gene function hypotheses and the identification of genes with similar roles (Warde-Farley et al., 2010). The integration algorithm of this network employs various bioinformatics approaches, such as physical interaction, co-expression, predicted, co-localization, genetic interactions, pathway, and shared protein domains. The IMvigor210 cohort consists of 298 patients with urothelial carcinoma who received atezolizumab (anti-PDL1), while the GSE91061 cohort includes 51 patients with melanoma who were treated with nivolumab (anti-PD1) prior to treatment. The data for the IMvigor210 cohort was obtained from http://research-pub.gene.com/IMvigor210CoreBiologies/packageVersions/, and the data for the GSE91061 cohort was acquired from the Gene Expression Omnibus database (https://www.ncbi.nlm.nih.gov/geo/). The abbreviations of cancers are provided in Table 1.

**TABLE 1:** TCGA cancer abbreviations and the corresponding cancer type.

Abbreviations	Cancer type
ACC	Adrenocortical carcinoma
BLCA	Bladder Urothelial Carcinoma
BRCA	Breast invasive carcinoma
CESC	Cervical squamous cell carcinoma and endocervical adenocarcinoma
CHOL	Cholangiocarcinoma
COAD	Colon adenocarcinoma
DLBC	Lymphoid Neoplasm Diffuse Large B- cell Lymphoma
ESCA	Esophageal carcinoma
GBM	Glioblastoma multiforme
HNSC	Head and Neck squamous cell carcinoma
KICH	Kidney Chromophobe
KIRC	Kidney renal clear cell carcinoma
KIRP	Kidney renal papillary cell carcinoma
LAML	Acute Myeloid Leukemia
LGG	Brain Lower Grade Glioma
LIHC	Liver hepatocellular carcinoma
LUAD	Lung adenocarcinoma
LUSC	Lung squamous cell carcinoma
MESO	Mesothelioma
OV	Ovarian serous cystadenocarcinoma
PAAD	Pancreatic adenocarcinoma
PCPG	Pheochromocytoma and Paraganglioma
PRAD	Prostate adenocarcinoma
READ	Rectum adenocarcinoma
SARC	Sarcoma
SKCM	Skin Cutaneous Melanoma
STAD	Stomach adenocarcinoma
TGCT	Testicular Germ Cell Tumors
THCA	Thyroid carcinoma
THYM	Thymoma
UCEC	Uterine Corpus Endometrial Carcinoma
UCS	Uterine Carcinosarcoma
UVM	Uveal Melanoma

### 2.2 Single-cell analysis of ABI3

The TISCH web tool (http://tisch1.comp-genomics.org/) (Sun et al., 2021) was leveraged for the single-cell analysis, with the following analytical settings: ABI3 (Gene), major lineage (Cell-type annotation), and all cancers (Cancer type). Levels of ABI3 expression were assessed and visualized in a heatmap for each cell type.

### 2.3 Survival and prognostic analysis of ABI3 in pan-cancer

PanCanSurvPlot (https://smuonco.shinyapps.io/PanCanSurvPlot/) is a web-based platform that utilizes a commonly adopted preparation method to scrutinize transcription data from both TCGA and Gene Expression Omnibus (GEO). The platform utilizes Cox regression analysis to determine the correlation between ABI3 expression and patients’ survival rates, including overall survival (OS), disease-specific survival (DSS), disease-free interval (DFI), and progression-free survival (PFS). The analysis is performed using integrated data sourced from TCGA databases available on the platform’s website. For evaluating the prognostic potential of ABI3, the platform selected the IlluminaHiSeq platform and the best cutpoint grouping method tailored to the specific prognosis type for each cancer. Using the R packages “forestplot,” the results were presented in the form of hazard ratios (HR) accompanied by 95% confidence intervals (95% CI), which were calculated as part of the analysis.

### 2.4 Cancer-associated genomic alteration and mutation profile of ABI3

To analyze the frequency of four genomic alteration types (mutation, amplification, deep deletion, and multiple alterations) in neoplasms, the Cancer Types Summary module of the web tool cBioPortal (https://www.cbioportal.org/) (Tuck and Janssens, 1990) was leveraged. The GSCA platform, a web server that integrates multiomics data based on the TCGA database (http://bioinfo.life.hust.edu.cn/GSCA) (Liu et al., 2018), was employed to investigate the distribution of ABI3 gene CNV, the association between mRNA expression levels of ABI3 and CNV, and associated survival differences between the CNV and wild type of the ABI3 gene across cancers. Additionally, GSCA was leveraged to scrutinize the methylation differences between normal and tumor samples of the ABI3 gene across various cancer types, the relationship between mRNA expression levels and the degree of methylation of ABI3 across cancers, and the associated survival differences between the higher and lower methylation groups of the ABI3 gene in these cancers. Spearman correlation analysis was used to assess the relationship of ABI3 gene mRNA expression with CNV or methylation levels, and the *p*-values were adjusted using the false discovery rate (FDR). Differential methylation across cancers was estimated using a *t*-test, and the *p*-values were further adjusted by FDR. To determine the statistical significance of the survival difference between groups, including OS, DSS, and PFS, a Logrank test was performed.

### 2.5 Immunotherapy prediction analysis

To calculate the TMB and MSI of each TCGA tumor case based on somatic mutation data obtained from the TCGA database (https://tcga.xenahubs.net), the R package “maftools” was used. The association between ABI3 expression and TMB, as well as MSI, was analyzed using Spearman’s rank correlation coefficient. The results were presented using a radar map generated with the R-package “ggradar.” The immunotherapeutic approaches were classified into four outcomes: progressive disease (PD), and stable disease (SD), complete response (CR) and partial response (PR). The optimal cutoff value was calculated based on the patients’ survival data using the “survminer” package in R, and the two relevant independent immunotherapeutic cohorts were separated into low-ABI3 and high-ABI3 groups. The survival and responder status of each group were then calculated separately. The levels of the MMR genes, including MutL Homolog 1 (MLH1), MutS Homolog 2 (MSH2), MutS Homolog 6 (MSH6), PMS1 Homolog 2 (PMS2), and Epithelial Cell Adhesion Molecule (EPCAM), were evaluated in different cancers through expression profile data from TCGA. The relationship between the expression levels of MMR genes and ABI3 was determined. Heatmap was generated to visualize these findings through the R-packages “tidyverse” and “ggnewscale.”

### 2.6 Assessing the impact of ABI3 expression on immunity

Estimation of Stromal and Immune Cells in Malignant Tumor Tissues Using Expression Data (ESTIMATE) method utilizes gene expression profiles to evaluate the extent of infiltration of immune or stromal cells within neoplasms (Yoshihara et al., 2013). Through the R packages “limma” and “estimate,” the association between ABI3 expression and the immune, stromal, and ESTIMATE scores, which are associated with the level of immune infiltration, was evaluated for each tumor sample. The illumina platform was used. The extent of immune cell infiltration in diverse cancer types was evaluated through the application of TIMER2.0. Infiltration data were acquired and inspected to determine if there is a relationship between infiltration and ABI3 expression. In pan-cancer, different immune algorithms were used to analyze the associations between ABI3 expression and 21 immune cell subsets, including CD4^+^ T cells, cancer-associated fibroblasts (CAF), progenitors of lymphoid cells, progenitors of myeloid cells, progenitors of monocytes, endothelial cells (Endo), eosinophils (Eos), hematopoietic stem cells (HSC), T follicular helper cells (Tfh), γ/δ T cells, natural killer T cells (NK T cells), regulatory T cells (Tregs), myeloid-derived suppressor cells (MDSCs), B cells, neutrophils, monocytes, macrophages, dendriticcells, NK cells, Mast cells, and CD8^+^ T cells. TIMER2.0 has already performed calculations and recorded the immune cell infiltration scores for various cancers data sourced from the TCGA database. These data were collected and assessed to establish any potential association between ABI3 expression and infiltration. Additionally, 150 immune-related genes, including those encoding major histocompatibility complex (MHC), immunosuppressive, chemokine receptor, immune activation, and chemokine proteins, were downloaded from TISDB (Ru et al., 2019). The relationship between ABI3 expression and immune-related genes were calculated using Spearman’s rank correlation coefficient in R. The results were visualized using R-packages “limma,” “pheatmap,” and “ggplot2.”

### 2.7 The biological significance analysis of ABI3 expression

To evaluate the biological functions of ABI3 in tumors, GSEA and GSVA were performed. The R package “msigdbr” was adopted to directly collect the C2 and C5 gene sets from the Molecular Signatures Database for calculating the normalized enrichment score and FDR of differentially expressed genes between low- and high-ABI3 expression cancer groups for each biological process across various types of cancers. R-packages “tidyverse,” “limma,” “org.Hs.eg.db,” “gseaplot2,” and “clusterProfiler” (Wu et al., 2021) were used for functional analysis. Tumor samples of each type were categorized into high and low expression groups based on the median value of. ABI3 gene expression level using the R package “limma,” and GSVA scores were produced for all cancers. ABI3 expression was analyzed for its correlation with 186 Kyoto Encyclopedia of Genes and Genomes (KEGG) pathways and 10,402 Gene Ontology (GO) terms in each tumor, with visualization of the 15 pathways exhibiting the most noteworthy positive and negative correlations. Other R packages used for GSVA analysis include “GSVA,” “ggprism,” “GSEABase,” “ggthemes,” “BiocParallel,” “tidyverse,” and “clusterProfiler.”

### 2.8 Correlation of ABI3 expression with drug sensitivity

The CellMiner database (http://discover.nci.nih.gov/cellminer/) has indicated a potentially promising relationship between ABI3 expression and drug response. This database has been tailored to cater to the needs of cancer research experts, allowing them to effectively combine and scrutinize molecular and pharmacological information related to the NCI-60 cancer cell lines. These cell lines are the most extensively employed cancer samples for screening anti-cancer drugs (Reinhold et al., 2012).

To access the relevant data, the processed dataset, RNA expression data (RNA: RNA-seq), and drug data (compound activity: DTP NCI-60) should be sequentially selected on the CellMiner website, and then download. To filter out data related to drugs with an “FDA approved” or “Clinical trial” status, the R package “limma” can be used. In addition, data matrices with more than 80% missing column values should be eliminated. To handle missing data, the R package “Impute” can be utilized. Graphs can be generated using “ggplot2” and “ggpubr.” Statistical significance was set at a *p*-value < 0.05.

Specifically, Autodock4 software was used to perform molecular docking studies and evaluate the binding energy and interaction mode between the drug Megestrol acetate, identified from the CellMiner database, and the ABI3 protein (Morris et al., 2009). The PubChem compound database (https://pubchem.ncbi.nlm.nih.gov/) (Kim et al., 2021) was used to obtain the molecular structure of Megestrol acetate. The 3D structure of the ABI3 protein was predicted using AlphaFold (https://alphafold.ebi.ac.uk/) (Jumper et al., 2021), and the Pymol software 2.2 was employed to visualize the model. In addition, Proteins Plus (https://proteins.plus/) was utilized to create 2D depictions. To generate two-dimensional diagrams of complexes with known 3D structures based on chemical drawing conventions, PoseView was utilized (Stierand et al., 2006).

### 2.9 Statistical analysis

For the purpose of bioinformatic validation, the entire dataset underwent a filtration process that involved eliminating missing and duplicated results, followed by a log2(TPM +1) transformation of the TPM values. The Mann-Whitney *U* test (also known as the Wilcoxon rank sum test) was utilized to compare the expression levels of ABI3 in normal and tumor tissues to determine statistical significance. The Kruskal-Wallis method was used to analyze the expression of ABI3 in the CCLE database, originating from diverse tissue sources. ABI3 expression levels were compared between groups or tumor and normal tissues using either paired t-tests or unpaired t-tests, depending on whether the samples were paired or unpaired. Spearman correlation analysis was conducted to evaluate the statistical associations between ABI3 expression and other factors of interest. The R software (Version 4.2.1; https://www.R-project.org) was utilized for data analysis. Significance was defined as a *p*-value less than 0.05.

## 3 Results

### 3.1 Basic information of ABI3

**FIGURE 1:**
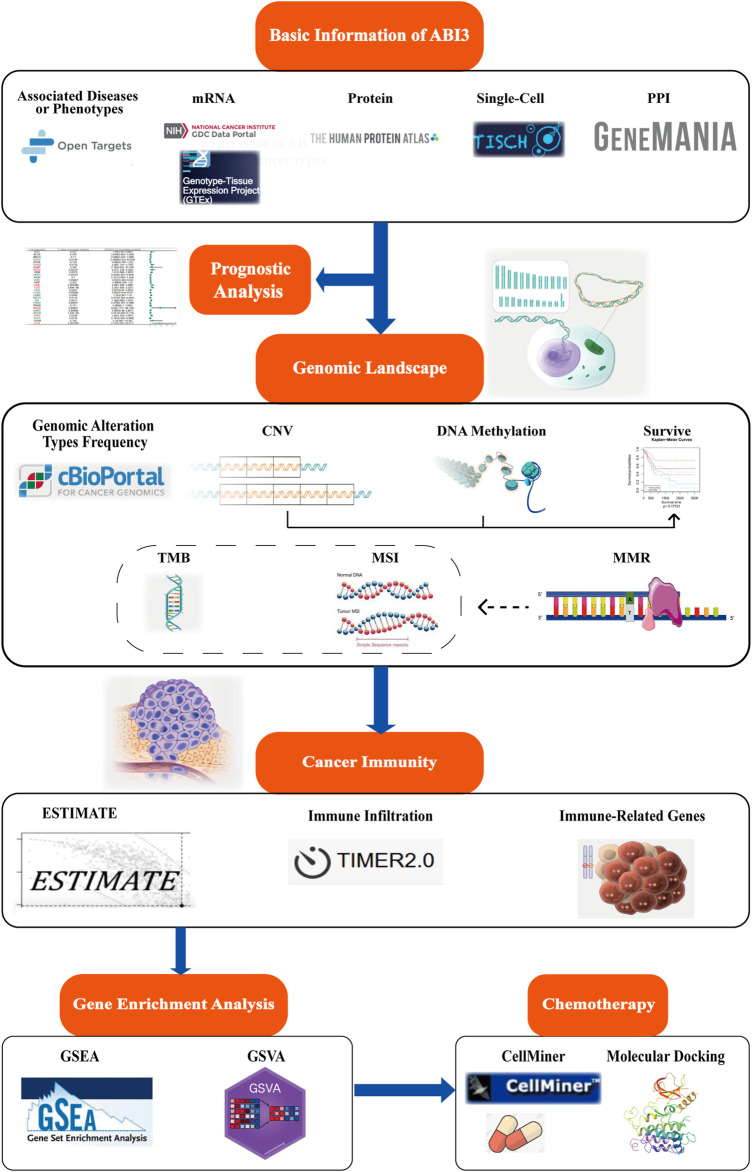
Schematic of this study design. The present study delves into the landscape of mRNA expression profiles, somatic mutation data, and clinical information, which were sourced from various databases and websites for follow-up analysis. The study investigates the expression variations between cancer and non-cancer tissues, as well as different cell types of ABI3. Patient survival data were grouped according to optimal segmentation points, and Cox proportional hazard regression analysis was performed. ABI3 expression association with genome instability was discovered through the cBioPortal database and GSCA web. Additionally, the clinical relevance of aberrant CNV and methylation was evaluated. Meanwhile, the correlation and significance of ABI3 expression with TMB, MSI, and other were compared among different cancer tissues. The correlation of ABI3 expression with ESTIMATE, immunocyte infiltration, and immune-related genes in diverse cancer types was visualized. This was further explored through the functional annotation profile, which pointed out the involvement of ABI3 in cancer immunity. Lastly, chemotherapeutic responses associated with ABI3 were predicted, and potential drugs underwent molecular docking analysis.

**FIGURE 2:**
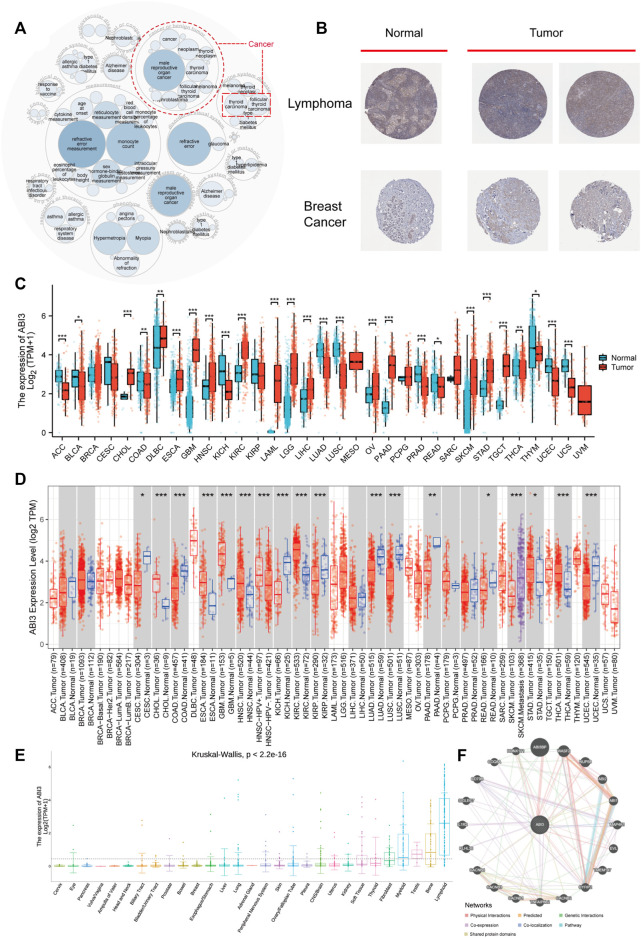
The expression ABI3 in human malignant cancers. **(A)** The diseases correlated with ABI3 were scrutinized using the openTarget online platform. The dashed red lines denote ABI3-correlated cancers. **(B)** Comparison of ABI3 protein expression according to immunohistochemical staining between normal (left) and tumorous (right) tissues in lymph nodes and breast. **(C)** By combining data from TCGA and GTEx, ABI3 expression divergences between tumor and standard tissues for 33 cancers were evaluated. **(D)** The state of ABI3 mRNA expression across cancers, and their corresponding control tissues were examined using the TIMER2.0 platform. The neoplastic and normal tissues were color-coded red and blue, respectively, while the SKCM metastatic tissues were represented in purple. **(E)** Using only CCLE data, ABI3 expression dissimilarities in 29 tissues were scrutinized based on their originating tissues. **(F)** The PPI network reveals the potential proteins that may interact with ABI3. Lines in different colors signifies the prediction methods.

Figure 1 provides an synopsis of the study’s comprehensive scope, and Table 1 outlines the complete abbreviations and names of the 33 cancers included in the analysis. To investigate ABI3-related diseases, we utilized OpenTarget and discovered that ABI3 was associated with thyroid carcinoma, as shown in the bubble graph in Figure 2A. In the HPA database, protein expression of ABI3 was only examined for two types of cancer. As depicted in Figure 2B, the IHC staining of ABI3 was observed to be moderate in normal lymph node tissues, whereas it was found to be strong in tumor tissues. On the other hand, weak ABI3 staining was detected in normal breast tissue samples, whereas moderate staining was observed in tumor tissues.

Regarding transcription, we initially utilized the TCGA and GTEx databases to investigate ABI3 expression levels in neoplasms compared to their corresponding normal tissues. As demonstrated in Figure 2C, ABI3 expression was significantly higher in CHOL, DLBC, ESCA, GBM, HNSC, KIRC, LAML, LGG, LIHC, OV, PAAD, SKCM, STAD, and TGCT than in the corresponding normal tissues. Conversely, ABI3 expression was lower in ACC, BLCA, COAD, KICH, LUAD, LUSC, PRAD, READ, THCA, THTM, UCEC, and UCS.

Subsequently, we employed TIMER2.0 to analyze ABI3 mRNA level differences between cancer and adjacent normal tissues. As illustrated in Figure 2D, ABI3 mRNA was remarkably augmented in 9 cancer types (CESC, COAD, KICH, KIRP, LUAD, LUSC, PAAD, READ, and UCEC) and downregulated in CHOL, ESCA, GBM, HNSC, KIRC, STAD, and THCA. Additionally, we downloaded the Expression Public 22Q4 file of ABI3 from the CCLE database to examine differences in ABI3 expression levels in 29 tissues based on the tissue source of the cancer cells. Our findings indicate that ABI3 is broadly dispersed in tissues, with particularly high expression levels in lymphoid tissues, as depicted in Figure 2E.

Lastly, we conducted a PPI network analysis according to interaction data acquired from the GeneMANIA website. The analysis identified 20 ABI3-related proteins derived from physical interaction, co-expression, predicted interaction, co-localization, genetic interactions, pathway analysis, and shared protein domains. As evident in Figure 2F, ABI3 had strong physical interactions with ABI3BP, WASF2, RUNX1T1, and other genes. It also showed co-expression with TNFAIP8L2 and SIGLEC7, and shared protein domains with ABI1 and ABI2. ABI3BP is a potential partner of ABI3, and previous studies have shown that their expression levels are synchronized during cancer progression (Latini et al., 2011). Moreover, there is existing literature demonstrating the important roles of ABI3BP, WASF2, RUNX1T1, and TNFAIP8L2 in cancer processes through pan-cancer analysis (Bai et al., 2022; Lin, 2022; Yang et al., 2022; Feng et al., 2023). This analysis suggests that ABI3 may influence cancer progression by interacting with the aforementioned genes.

### 3.2 Single-cell analysis revealed that ABI3 exhibits the highest expression abundance in monocyte/macrophage and Tprolif cells within TME

**FIGURE 3:**
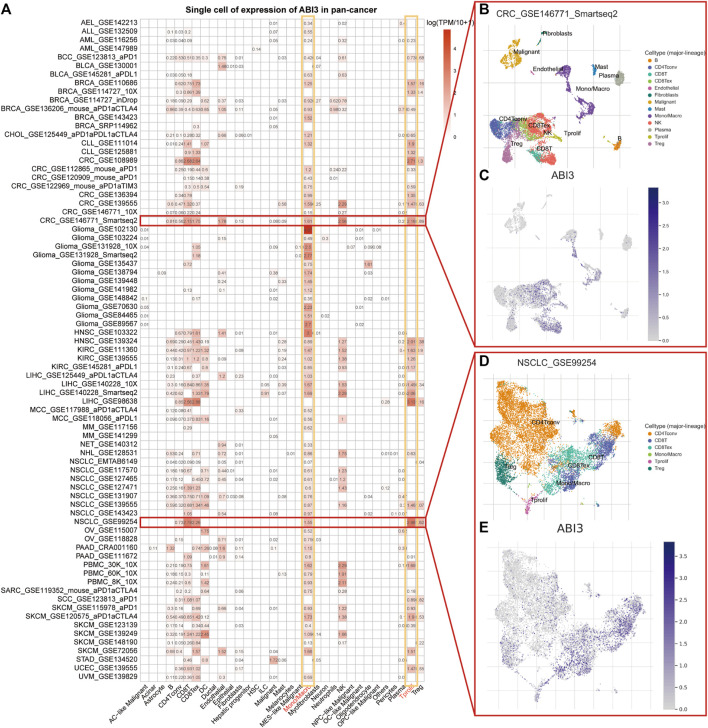
**(A)** Summary of ABI3 expression of 33 cell types in 77 single cell datasets. **(B)** A scatter plot showed the distributions of 13 distinct cell types of the GSE146771 CRC dataset. **(C)** A scatter plot showed the levels of ABI3 expression of cells in the GSE146771 CRC dataset. **(D)** A scatter plot showed the distributions of 6 different cell types of the GSE99254 NSCLC dataset. **(E)** A scatter plot showed the levels of ABI3 expression of cells in the GSE99254 NSCLC dataset.

To determine the primary cell types expressing ABI3 in tumor microenvironment (TME), we conducted a single-cell analysis of ABI3 in 77 single-cell datasets of cancer samples. Utilizing the TISCH web tool, we generated a heatmap of the expression levels of ABI3 in 33 cell types, including immune cells, functional cells, malignant cells, and stromal cells, as depicted in Figure 3A. Our findings elucidated that ABI3 was predominantly detected in immune cells, particularly in monocyte/macrophage and Tprolif cells.

Notably, in the GSE146771 dataset encompassing 10 primary colorectal cancer patients and comprising 10,468 cells, ABI3 expression was widely distributed across various immune cell types, such as T cell isoforms, endothelial, NK cells, monocytes, and macrophages in the colorectal cancer (CRC) microenvironment, as illustrated in Figures 3B, C. Similarly, in the GSE99254 dataset, we analyzed 12,346 cells from 14 non-small cell lung cancer (NSCLC) patients and found that ABI3 was markedly expressed in various T cell isoforms and monocytes/macrophages in the NSCLC microenvironment, as shown in Figures 3D, E.

### 3.3 Prognostic prediction function of ABI3 among multiple cancer types

**FIGURE 4:**
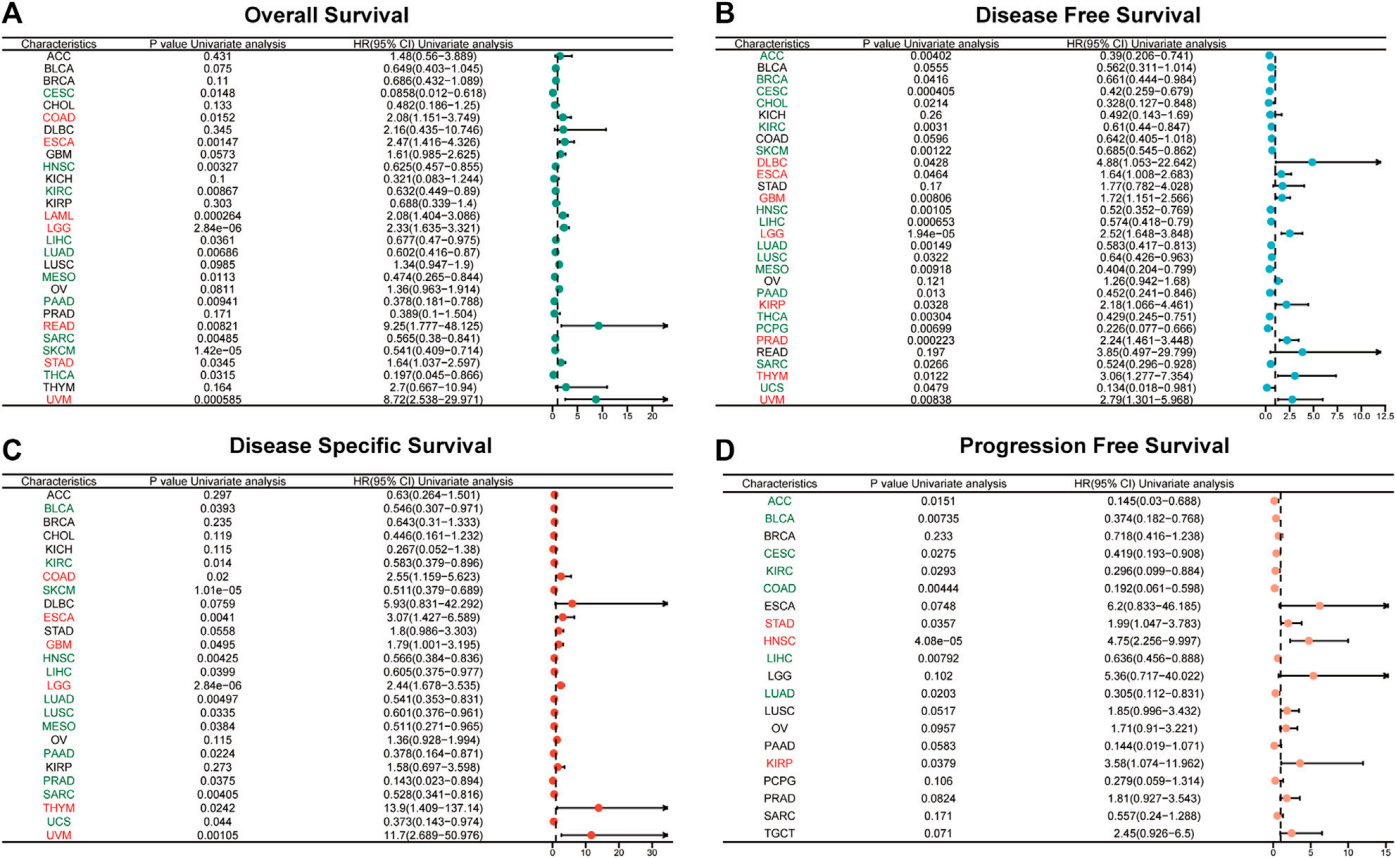
Univariate Cox regression analyses for the prognostic value of ABI3 expression in pan-cancer. Forest plot visualizing the association of ABI3 expression with OS **(A)**, DFS **(B)**, DSS **(C)**, as well as PFS **(D)** among pan-cancer. Hazard ratio (HR) value > 1 represents a risk factor (red), whereas HR value < 1 represents a favorable factor (green).

To investigate the correlation between ABI3 expression levels and prognosis, we conducted a survival analysis, including OS, DSS, DFI, and PFS, for each cancer type. Our pan-cancer prognosis analysis unveiled that ABI3 was closely tied to the prognosis of most cancers, with the exception of KICH and OV, as depicted in Figure 4.

Using the Cox proportional hazards model, we examined the prognosis across pan-cancer. As illustrated in Figure 4A, the forest plot results indicated that a positive correlation was evident between OS and ABI3 expression in COAD, ESCA, LAML, LGG, READ, STAD, and UVM, while a negative correlation was observed in CESC, HNSC, KIRC, LIHC, LUAD, MESO, PAAD, SARC, SKCM, and THCA. Regarding ABI3 expression and DFS, a markedly positive relationship was observed in DLBC, ESCA, GBM, LGG, KIRP, PRAD, THYM, and UVM. Additionally, we noted that ABI3 expression had a protective influence on DFS in ACC, BRCA, CESC, CHOL, KIRC, SKCM, HNSC, LIHC, LUAD, LUSC, MESO, PAAD, THCA, PCPG, SARC, and UCS, as shown in Figure 4B. As OS includes many noncancer deaths, we further conducted DSS analysis, which was more relevant to the effectiveness of cancer treatment. Our DSS analysis revealed that ABI3 expression had a favorable influence on BLCA, KIRC, SKCM, HNSC, LIHC, LUAD, LUSC, MESO, PAAD, PRAD, SARC, and UCS, but was identified as a hazard factor in COAD, ESCA, GBM, LGG, THYM, and UVM, as depicted in Figure 4C. Furthermore, according to the forest plot for PFS in Figure 4D, ABI3 expression was found to be a risk factor in STAD, HNSC, and KIRP, but a protective factor for patients with ACC, BLCA, CESC, KIRC, COAD, LIHC, and LUAD.

### 3.4 ABI3 gene alterations correlate with genomic instability and aberrations in pan-cancer

**FIGURE 5:**
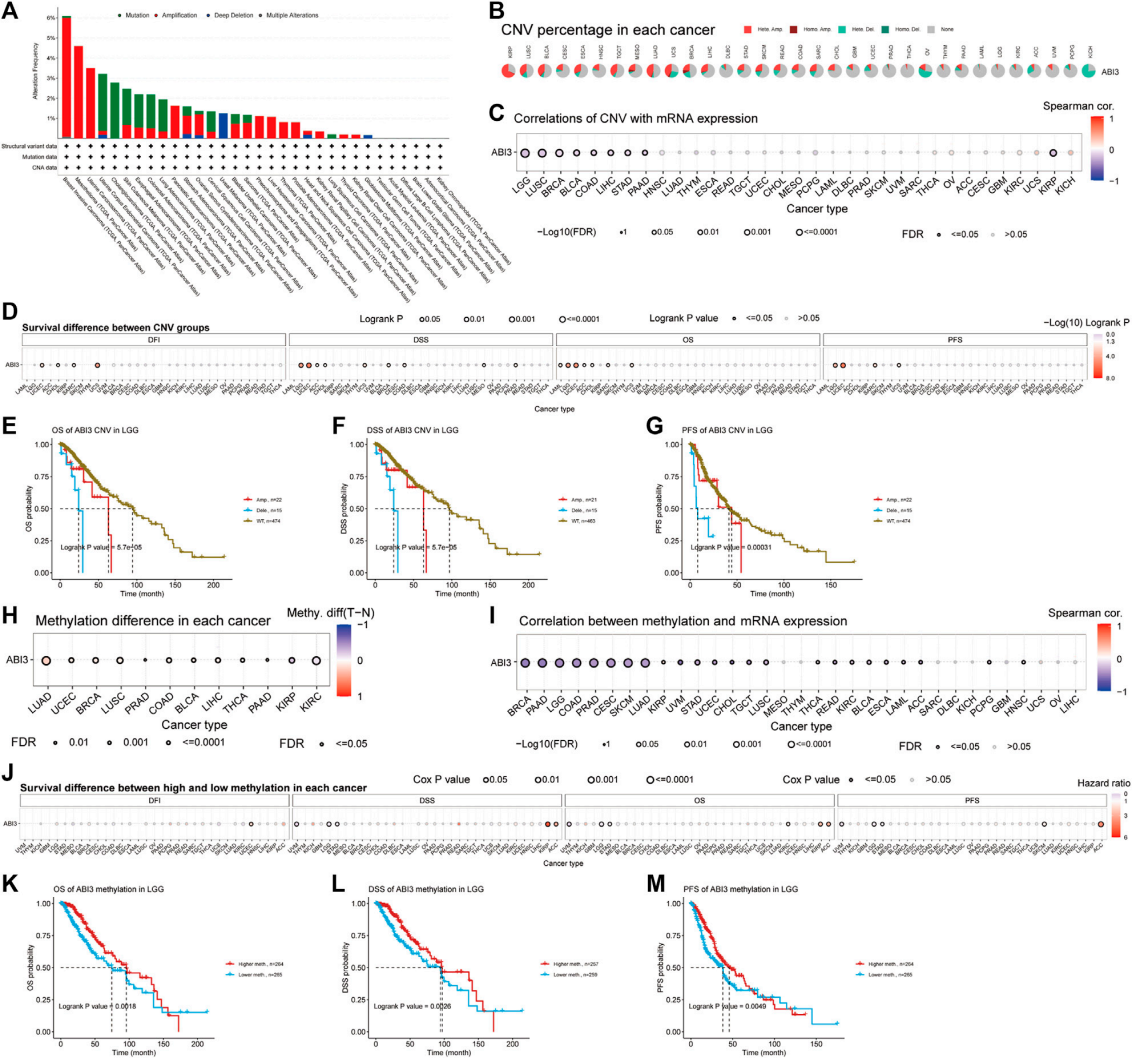
Genomic instability was found to be associated with ABI3 in TCGA tumors. **(A)** The genomic alterations of ABI3 were examined in the TCGA pan-cancer cohort, encompassing various alterations such as mutation, amplification, deep deletion, and multiple modifications. **(B)** CNV summary presents the summary of ABI3 gene CNV in various cancer types. **(C)** The relationship between ABI3 CNV and mRNA expression through Spearman correlation analysis. **(D)** Correlation between ABI3 CNV status and OS, DSS, PFS, and DFI in pan-cancer. **(E–G)** Kaplan-Meier survival curves were generated using the GSCA web tool to assess the prognostic value of ABI3 CNVs in patients diagnosed with LGG. **(H)** Differential analysis of methylation between tumor and normal sample groups was analyzed for cancer which have >10 pairs of tumor-normal samples. Only the results with *p*-value ≤ 0.05 are presented. **(I)** A Spearman correlation analysis was conducted to investigate the relationship between ABI3 methylation and mRNA expression in a pan-cancer cohort. **(J)** Correlation between ABI3 methylation status and OS, DSS, PFS, and DFI in pan cancer. **(K–M)** Kaplan-Meier survival curves were applied to depict the survival outcomes of LGG patients stratified according to their high- or low-ABI3 methylation status.

Cancers are characterized by genomic alterations. We examined the genomic alterations in the ABI3 gene across different cancer types through the cBioPortal database. Our analysis revealed that the frequency of ABI3 alterations varied across pan-cancer, with the highest prevalence of copy number alterations observed in BRCA, MESO, and UCS, where the majority of cases were amplifications (Figure 5A).

Additionally, we present the outcomes of the ABI3 pan-cancer CNV and methylation analysis in the GSCA database. The pie chart shows a global profile that displays the constitution of the heterozygous/homozygous CNV of the ABI3 gene in each cancer, with different colors representing different CNV types. The corresponding legend represents the proportion of samples with copy number heterozygous amplification, heterozygous deletion, homozygous amplification, homozygous deletion, or none. Furthermore, the bubble diagram shows the association between CNV and mRNA expression through correlation analysis. We discovered that the level of CNV and ABI3 mRNA expression presented a negative relation in patients with LGG, LUSC, BRCA, KIRP, BLCA, LIHC, STAD, COAD, and PAAD, as shown in Figures 5B, C. We then divided the samples into WT, Amp., and Dele. groups and examined the survival differences between CNV and the wide type of ABI3 gene in each cancer. After applying CNV levels for patient grouping by GSCA, our analysis revealed that high ABI3 CNV group patients had lower overall survival rates in several cancer types, including ACC, CHOL, LGG, LAML, SARC, UCEC, and UCS, as shown in Figure 5D. Notably, high ABI3 CNV group patients in LGG consistently showed worse prognoses across various survival indicators (Figures 5E–G).

DNA methylation is a chemical modification of DNA that can result in the inactivation of tumor suppressor genes and may be carcinogenic (Kulis and Esteller, 2010). Our analysis of methylation differences between normal and tumor samples, as depicted in Figure 5H, revealed that methylation levels were higher in tumor samples than in normal samples in BLCA, BRCA, COAD, LIHC, LUAD, LUSC, PAAD, PRAD, THCA, and UCEC cancer types. However, methylation levels were higher in normal samples in KIRC and KIRP cancers. Furthermore, we revealed that DNA methylation was negatively correlated with ABI3 expression in several cancer types, including BRCA, CESC, COAD, LGG, LUAD, PAAD, PRAD, SKCM, STAD, LUSC, UVM, UCEC, TGCT, BLCA, ESCA, KIRC, READ, CHOL, ACC, LAML, PCPG, HNSC, and THCA, while it was positively associated with ABI3 expression in KIRP (Figure 5I). We then categorized tumor samples based on the median methylation levels, separating them into high and low methylation groups and found that high ABI3 methylation level patients had lower overall survival rates in ACC, KIRP, and KIRC, as well as lower survival rates in LGG, STAD, and UVM, as shown in Figure 5J. Of note, various analyses of survival indicators consistently revealed that LGG patients with low methylation levels of the ABI3 gene had a higher risk of mortality (Figures 5K–M).

### 3.5 Association between ABI3 and immunotherapy-related factors

TMB, which is determined by counting somatic non-synonymous mutations within the coding region of a tumor genome (Thorsson et al., 2018), and MSI, decided by counting overall mutation occurrences per million base pairs due to MMR deficiency (Bonneville et al., 2017), are both critical factors that impact the sensitivity of immune checkpoint inhibitors and have significant implications for patient prognosis and therapeutic responses (Choucair et al., 2020; van Velzen et al., 2020). MMR is essential for repairing DNA replication errors that occur during cell division. When MMR genes are downregulated or functionally impaired, somatic mutations can arise (Baretti and Le, 2018).

**FIGURE 6:**
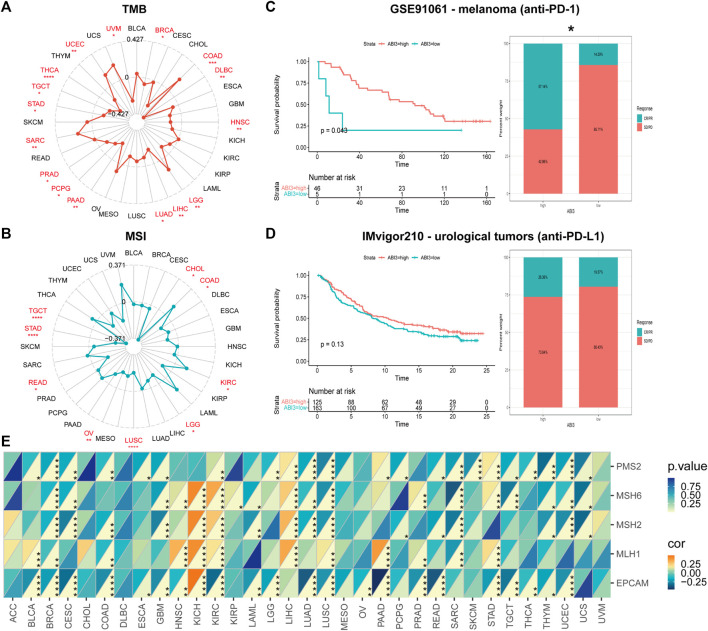
The correlation between ABI3 expression and TMB, MSI, and MMR across different cancer types was investigated. Radar charts ware employed to visualize the relationship between ABI3 expression and TMB **(A)** and MSI **(B)**. **(C,D)** The Kaplan-Meier curves and stacked bar chart depict the survival outcomes and the proportion of patients with response to immunotherapy for low- and high-PDIA3 patient groups in two distinct cohorts: GSE91061 (up) and IMvigor210 (down). **(E)** A heatmap was generated to display the correlation between ABI3 expression and five MMR genes in a pan-cancer cohort. Statistical significance was indicated as *p < 0.05, **p < 0.01, ***p < 0.001, and ****p < 0.0001.

As shown in Figures 6A, B, in four types of cancer (COAD, LGG, SARC, and UCEC), there was a positive correlation between ABI3 and TMB, and in two types of cancer (COAD and KIRC), there was a positive correlation between ABI3 and MSI. However, in twelve types of cancer (BRCA, DLBC, PAAD, PRAD, PCPG, LIHC, LUAD, HNSC, THCA, TGCT, STAD, and UVM), there was a negative correlation between ABI3 and TMB and, in seven types of cancer (CHOL, LGG, LUSC, READ, OV, TGCT, and STAD), there was a positive correlation between ABI3 and MSI.

Investigating ABI3 expression’s potential as a predictive biomarker for immunotherapy response, our analysis encompassed two cohorts, including anti-PD-1 and anti-PD-L1 therapies. We observed that melanoma patients with high-ABI3 expression demonstrated better survival probability and higher response rates (57.14%) to anti-PD-1 therapy (Figure 6C). In a comparable manner, an inclination in the IMvigor210 cohorts was discerned, wherein patients with elevated ABI3 expression appeared to demonstrate more responsive to immunotherapy (Figure 6D).

We next investigated the relationship between expression levels of MMR genes, including MLH1, MSH2, MSH6, PMS2, and EPCAM, and ABI3 expression level. As depicted in Figure 6E, MMR gene expression was significantly and negatively correlated with ABI3 levels in most tumors, except for KICH and LIHC. These findings strongly support the potential of ABI3 as a biomarker for genome stability in LGG.

### 3.6 Association between ABI3 expression and immune-related factors

**FIGURE 7:**
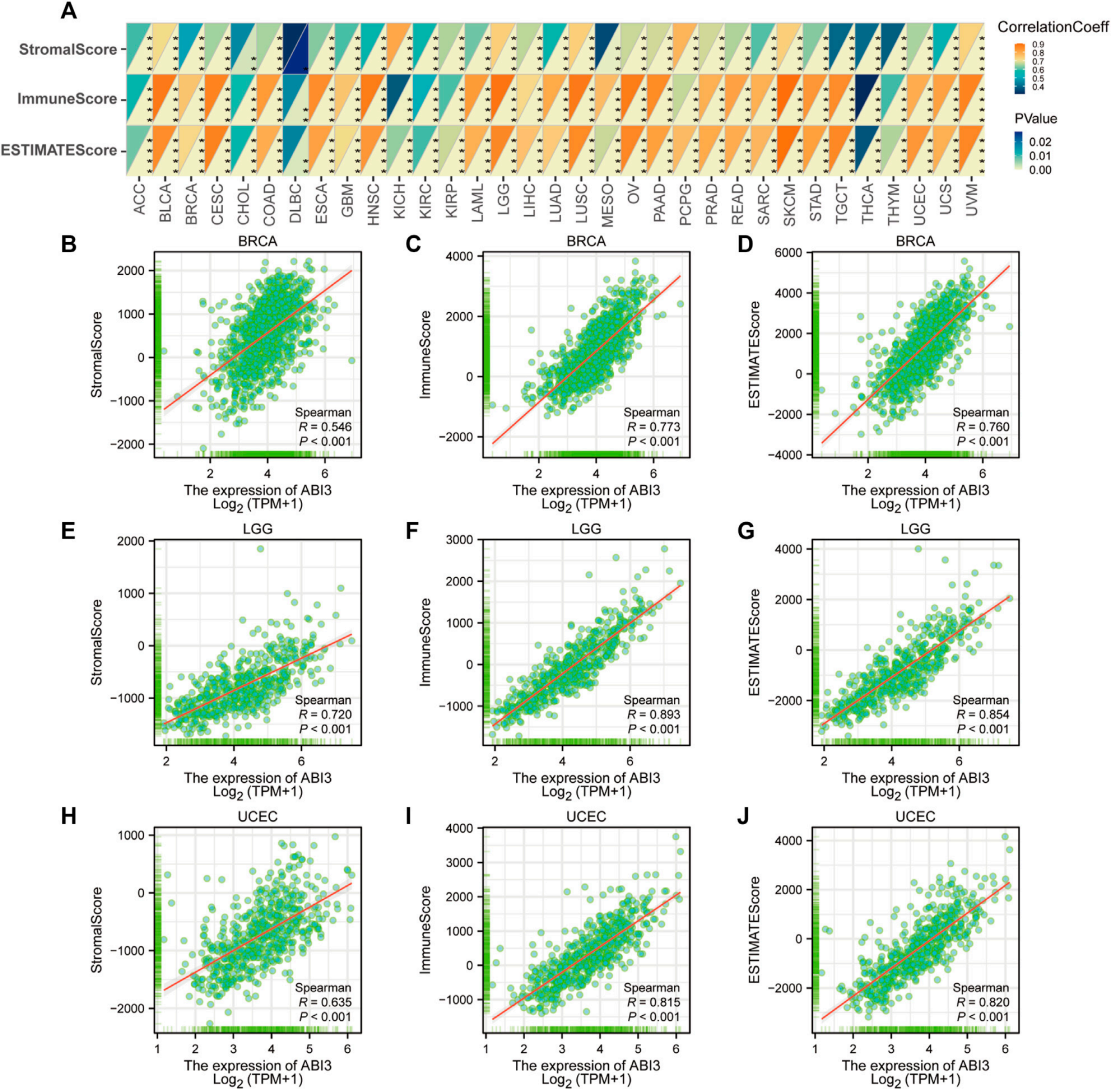
Analysis of ABI3 expression related immune characteristics. **(A)** A Spearman’s correlation analysis was conducted to investigate the relationship between ABI3 expression and ImmuneScore, StromalScore, and EstimateScore in a pan-cancer cohort. The correlation coefficient was represented in the top left triangle, while the *p*-value was represented in the bottom right triangle (*p < 0.05, **p < 0.01, ***p < 0.001, ****p < 0.0001). Correlation between ABI3 expression and three scores in BRCA **(B–D)**, LGG **(E–G)**, and UCEC **(H–J)**.

The TME constitutes an intricate assemblage, encompassing stromal cells, fibroblasts, endothelial cells, innate immune sentinels, in conjunction with adaptive immune actors. Deciphering the nuances of this milieu is imperative to optimizing therapeutic outcomes (Hinshaw and Shevde, 2019). To ascertain the nexus between ABI3 expression and facets of the tumor microenvirons, we gauged stromal indices, immune metrics, and ESTIMATE values transversely across neoplastic entities (Figure 7A). As portrayed in Figure 7A, ABI3 expression manifested a positive correlation with StromalScore, ImmuneScore, and EstimateScore in the preponderance of neoplasms with statistical significance. The outcomes for BRCA, LGG, and UCEC are visualized and depicted in granular detail in Figures 7B–J.

### 3.7 Association between ABI3 expression and immune infiltration/immunoregulation genes

**FIGURE 8:**
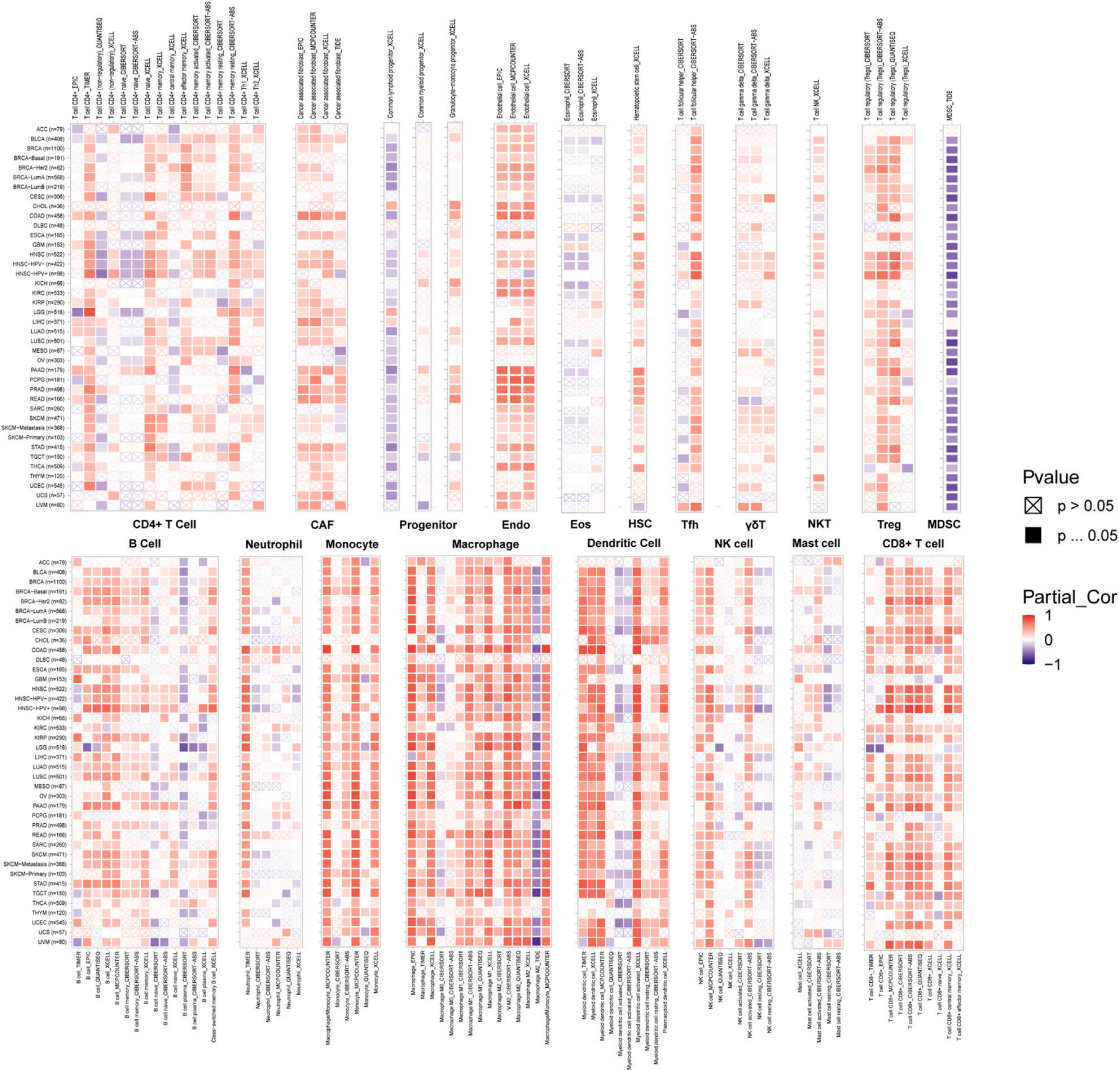
A strong connection between ABI3 expression and the degree of infiltration levels of CD4^+^ T cells, CAF, progenitor, Endo, Eos, HSC, Tfh, gdT, NKT, Tregs, B cells, neutrophils, monocytes, macrophages, dendritic cells, NK cells, Mast cells and CD8^+^ T cells in multiple malignancies accessible in the TIMER 2.0 database and obtained from TCGA. The color red was used to denote a positive correlation, while the color blue was used to indicate a negative correlation.

To delineate the interconnections between ABI3 and oncological immunity, we probed deeper into the affiliations between immune cell infiltrations and ABI3 expression. We implemented Spearman correlation analyses exploiting the tumors immune cell infiltration data from the TIMER2.0 repository. Our findings unveiled the infiltration levels of diverse immune cell types, such as CD4^+^ T cells, CAFs, progenitors of lymphoid and myeloid cells, progenitors of monocytes, Endo, Eos, HSCs, Tfh, γ/δ T cells, NK T cells, Tregs, myeloid-derived suppressor cells (MDSCs), B cells, neutrophils, monocytes, macrophages, dendritic cells, NK cells, mast cells, and CD8^+^ T cells across pan-cancer specimens (Figure 8). Our results demonstrated that ABI3 expression manifested a positive correlation with the infiltration levels of CD4^+^ T cells, CAFs, Endo, HSCs, γ/δ T cells, NK T cells, Tregs, B cells, monocytes, and CD8^+^ T cells in the majority of the TCGA cancers. Conversely, ABI3 expression manifested a negative association with the infiltration levels of progenitors of lymphoid and MDSCs in majority TCGA cancers, especially in BRCA and UCEC. Recent studies have accentuated the pivotal role of immune cells, such as CD4^+^ T cells, CAFs, MDSCs, neutrophils, and macrophages, in oncological immunotherapy (Borst et al., 2018; Galbo et al., 2021). Therefore, the significance of immune cells in tumor management cannot be overemphasized. Our results imply that ABI3 potentially impacts the tumorigenesis, prediction, and antineoplastic therapy by interacting with immune cells.

**FIGURE 9:**
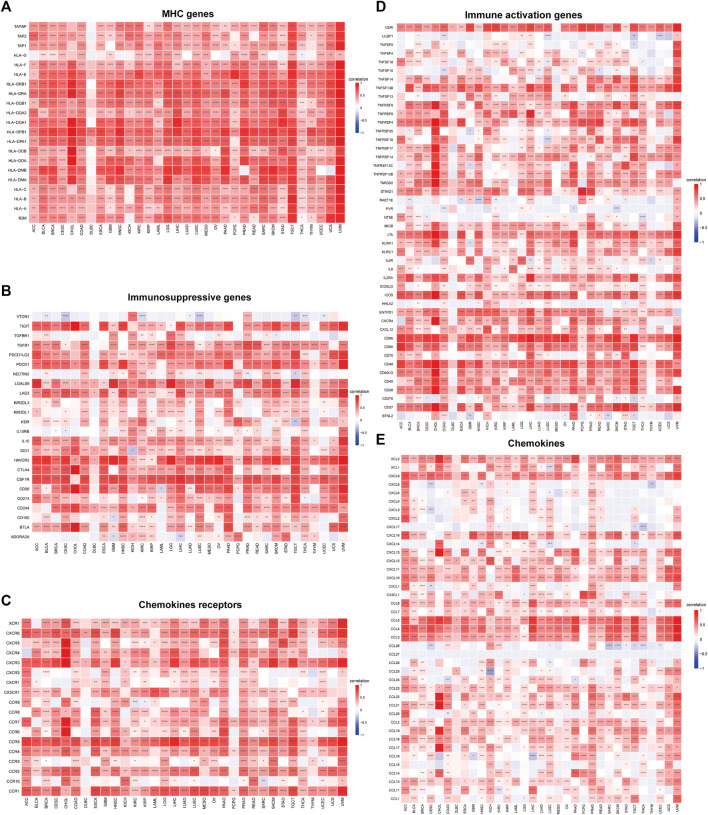
Relationship between ABI3 expression and **(A)** chemokine receptors, **(B)** chemokines, **(C)** immunosuppressive factors, **(D)** immunostimulatory factors, and **(E)** MHC genes. *p < 0.05, **p < 0.01, ***p < 0.001, ****p < 0.0001.

Moreover, we executed gene expression co-regulation analysis to investigate the connections between ABI3 expression and immunoreactive genes in 33 tumors. The examined genes encoded MHC proteins, immune activation and immunosuppressive proteins, chemokines, and chemokine receptor proteins. The generated heatmap demonstrated that practically all immunoreactive genes were co-expressed with ABI3 (Figure 9), and the plurality of these genes exhibited a strong and positive association with ABI3 in all tumor types, with the exception of DLBC.

### 3.8 The biological significance of ABI3 expression in neoplasms

To investigate the functional roles of ABI3 in cancers, we performed sequential functional enrichment analyses. The samples were stratified based on the median ABI3 expression in each cancer type, and bifurcated into low and high-ABI3 groups for GSEA and GSVA to discern the biological processes associated with ABI3.

**FIGURE 10:**
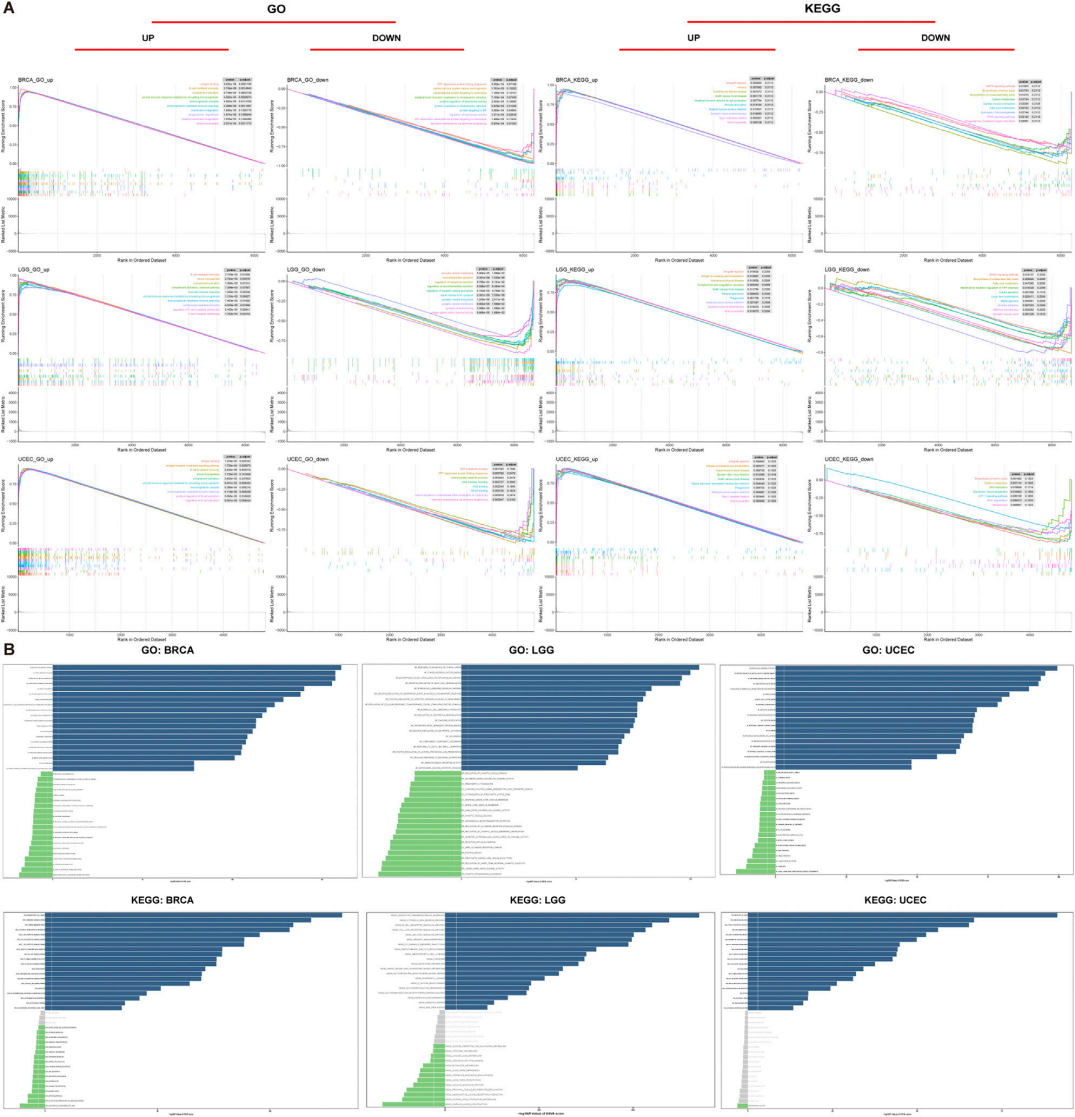
Functional analyses of ABI3. **(A)** GO functional annotation and KEGG pathway analysis of GSEA of ABI3 in BRCA, LGG, and UCEC. Curves of varying colors depict diverse functions or pathways that are regulated in different types of cancers. Peaks on the ascending curve signify positive regulation, whereas peaks on the descending curve indicate negative regulation. **(B)** GSVA results were obtained using GO and KEGG datasets across BRCA, LGG, and UCEC. Pathways with the most considerably positive correlation were represented using slate-colored bars. Green bars show pathways with the most significant negative correlations. Gray bars represent insignificant pathways, where the FDR is greater than 0.05. The horizontal axis represents -log10(*p*.value) of GSVA score.

We discovered that ABI3 positively regulates a range of immune-related activities in BRCA, LGG and UCEC, including B cell mediated immunity, humoral immune response mediated by circulating immunoglobulin, immunoglobulin mediated immune response, and other GO terms or allograft rejection, Autoimmune thyroid disease, Graft−versus−host disease, and other KEGG pathways. These findings revealed a possible nexus between the expression of ABI3 and immune activation within the TME. In contrast, ABI3 was predicted to be a negative regulator of protein synthesis and telomerase activity in BRAC and UCEC, synaptic vesicle-related activities in LGG, biological processes such as the activity of DNA and the degradation of RNA in UCEC (Figure 10A).

In addition, we utilized GSVA to further investigate the biological implications of ABI3 expression in the aforementioned tumors. Figures 10B, C displays the top 15 pathways that exhibited a remarkable positive or negative association with ABI3 expression in these three neoplasms. Our findings revealed that ABI3 expression was positively pertained to various immune-related pathways, such as negative regulation of neutrophil activation, immunoglobulin receptor activity, and pathways associated with immune cells including B and T cells, neutrophils, and lymphocytes. Furthermore, we observed several immune factor-related pathways, such as chemokine, interleukin 15, TNF, and synaptic pruning. By contrast, ABI3 expression was inversely associated with energy metabolism-related pathways in BRCA, telomerase activity-related pathways in LGG (such as regulation of synaptic vesicle membrane organization), and cell cycle-related pathways in UCEC.

### 3.9 ABI3 and drug response

**FIGURE 11:**
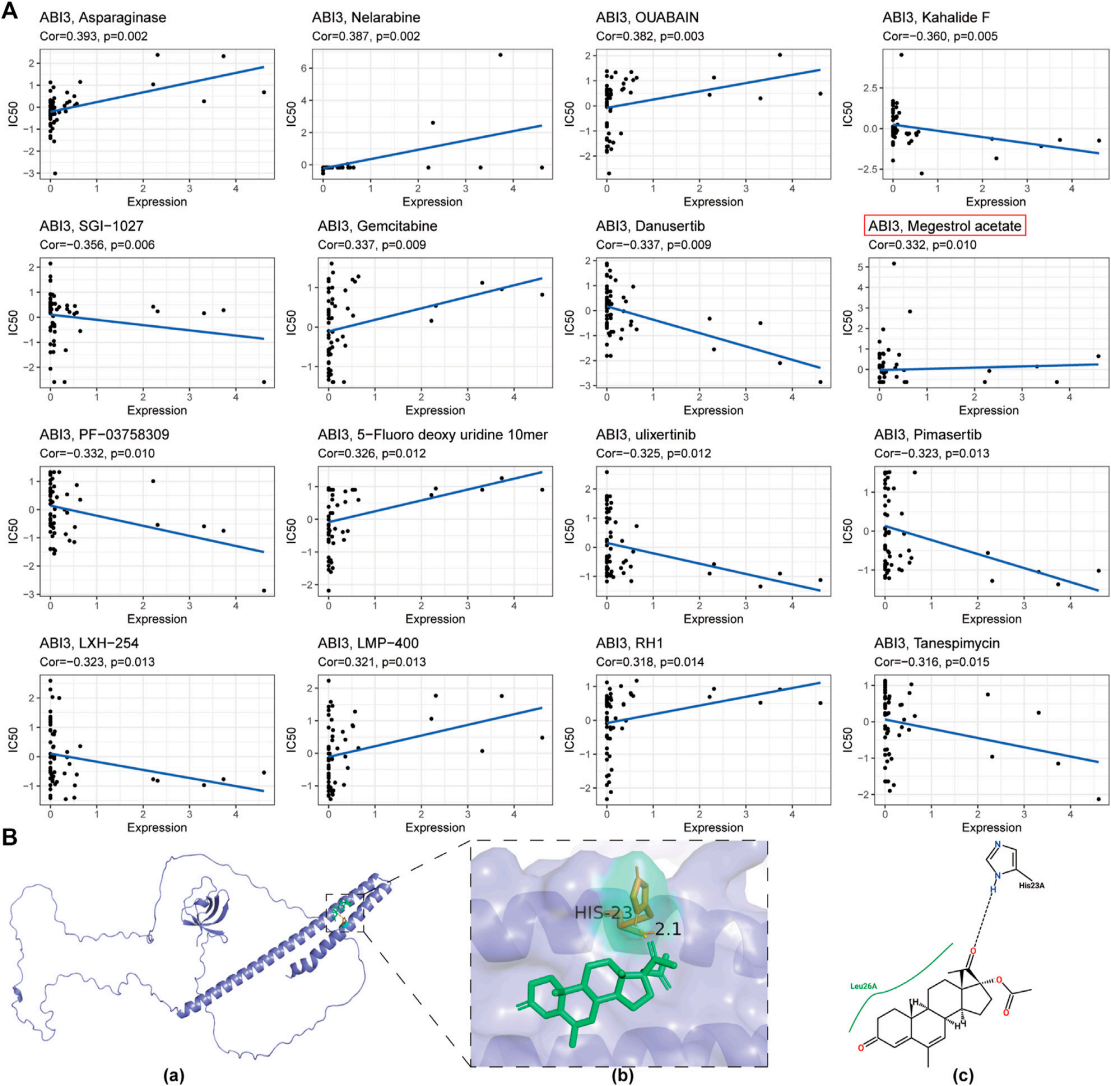
**(A)** A depiction of the correlation between ABI3 expression and anticipated drug response obtained from the NCI-60 cancer cell line panel. **(B)** The predicted interaction of Megestrol acetate with ABI3 protein. (a) The ribbon structure of ABI3 protein was depicted and Megestrol acetate was shown in a stick representation. (b) A close-up view of the interactions of Megestrol acetate with ABI3 protein. Important receptor residues that contribute to interaction are shown in a stick representation and labeled. The receptor was represented using slate cartoon renderings. Sticks represent Megestrol acetate (green) as well as receptor residues (orange) involved in ligand binding. (c) The 2D hydrogen bond interaction pattern of Megestrol acetate upon binding to ABI3 protein.

Figure 11A displays the drugs that exhibited the most significant correlations, indicating a positive relationship between drug response and ABI3 expression in patients treated with Asparaginase, Nelarabine, OUABAIN, Gemcitabine, Megestrol acetate, 5-Fluoro deoxy uridine 10mer, LMP-400, and RH1. In contrast, there was a negative correlation between ABI3 expression and several anticancer drugs, including Tanespimycin, LXH-254, Pimasertib, Ulixertinib, PF-03758309, Danusertib, SGI-1027, and Kahalide F.

To evaluate the affinity of drugs selected by the CellMiner database for the ABI3 protein, we performed molecular docking analysis using Autodock4 software to obtain the binding pose and interactions between the drug and protein, as well as the binding energy for each interaction. Our results revealed that Megestrol acetate bound to the ABI3 protein through visible hydrogen bonds and strong electrostatic interactions (Figure 11B). The best pose offered a calculated binding energy of −5.3 kcal/mol, indicating highly stable binding.

## 4 Discussion

Cancer immunotherapy has revolutionized treatment options for patients, with immune checkpoint blockade representing a groundbreaking approach (Wang et al., 2019). However, the heterogeneity of the tumor microenvironment limits the efficacy of immunotherapy, resulting in only a segment of cancers and a minute proportion of patients exhibiting favorable responses. Thus, identifying new and precise biomarkers to improve patient outcomes is an urgent need.

ABI3 may play a unique role as a critical regulator of Rac-mediated actin polymerization, which is involved in cellular motility and impacts cancer aggressiveness and metastasis. Unrestrained proliferation and invasion are the most hazardous pathological changes underlying the complexity and enigmatic nature of all malignancies (Evan and Vousden, 2001). The significance of ABI3 in cellular motility was noteworthy, which was why we are focusing on this gene. In this investigation, we utilized publicly-funded cancer genomics programs and repositories to explore the landscape of different tumor types based on ABI3 expression, with the aim of uncovering its potential role in tumorigenesis. Our results demonstrated that ABI3 served as a reliable prognostic biomarker for pan-cancer.

Initially, we scrutinized the expression level of ABI3 in neoplasms on the grounds of TCGA and GTEx data. Our findings were consistent with a previous study (Latini et al., 2011) indicating that ABI3 expression was downregulated in COAD and THCA. Nevertheless, we also identified that ABI3 was aberrantly upregulated in 14 types of cancer, which contradicts the previous notion that its expression was decreased in invasive malignancies, suggesting underlying functions of ABI3 across multiple cancer types.

Furthermore, ABI3 expression was found to be enriched in monocyte/macrophage and Tprolif cells in the immune microenvironment across various single-cell databases in the TISCH website. Interestingly, within the subtypes of Non-Small Cell Lung Cancer and Colorectal Cancer, ABI3 expression was most abundant within immune cells.

Following this, we investigated the relationship between ABI3 and cancer patient prognosis. Our analysis of various indicators, including OS, DSS, DFI, and PFS, consistently revealed that ABI3 was profoundly linked to cancer patient prognoses. Building on our previous ABI3 expression analysis, we observed that high levels of ABI3 expression were associated with worse prognosis in LGG and ESCA, but better prognosis in LIHC and KIRC. Prior research has highlighted the potential of ABI family members as cancer biomarkers, with ABI family member overexpression posing a risk factor in LIHC and colorectal cancer, while decreased expression being linked to worse prognosis in gastric cancer (Cui et al., 2010; Wang et al., 2017a; Zhang et al., 2021). Our findings suggest that ABI3 is critically involved in anticipating cancer patient survival rates and could perform as a reliable indicator for prognosis for cancer patients.

Subsequently, we investigated ABI3 alterations utilizing the cBioPortal repository and discovered that ABI3 manifested the highest prevalence of copy number alterations in patients with BRCA. It was worthwhile exploring the role of ABI3 gene variation, particularly copy number alterations, as an oncological sensor in BRCA patients. We thoroughly probed the relationships between CNV, methylation, and ABI3 expression. Utilizing the GSCA database, we found that ABI3 expression exhibited an inverse correlation with CNV and methylation levels in patients with LGG. Furthermore, individuals with aberrant copy number and hypomethylation of ABI3 had a worse prognosis. These findings imply that ABI3 was a promising prognostic biomarker for LGG and that DNA copy number variation and methylation may be two underlying drivers of ABI3 dysregulation in LGG.

TMB can also perform as a prognostic signature following immunotherapy in pan-cancer patients (Samstein et al., 2019) and has the potential to aid in the selection of immunotherapy in the age of precision medicine (Steuer and Ramalingam, 2018). MSI is associated with a heightened risk of malignancy with specific clinicopathological features, including elevated TMB and infiltration of lymphocytes into the malignancy, and is also a critical prognostic marker for PD-1 Blockade (Dudley et al., 2016). Our investigation demonstrated that ABI3 expression manifested a correlation with TMB in 16 cancer classifications and with MSI in 9 cancer classifications. This observation suggests that the expression level of ABI3 may impact the TMB and MSI of neoplasms, consequently impacting the patient’s reaction to immunotherapy utilizing immune checkpoint inhibitors. These findings imply that future research efforts should focus on modifying the TME or MSI by targeting ABI3, providing insight into the potential of ABI3 expression to predict the effect of immunotherapy and stratify patients for the selection of immune checkpoint blockade treatment. We examined the association between ABI3 expression and immunotherapeutic responsiveness, specifically in relation to immune checkpoint inhibitors like anti-PD-L1 and anti-PD-1 antibodies, by analyzing two pertinent cohorts, unveiling ABI3’s potential as a predictive marker for immune checkpoint blockade therapy sensitivity in cancer immunotherapy. We demonstrated ABI3 as a robust prognostic biomarker in melanomas, effectively predicting responses to anti-PD-1 immunotherapies. However, further studies on additional cohorts are needed to comprehensively evaluate ABI3’s value as an immunotherapy biomarker in cancer treatment. In most tumors, except for LIHC and KICH, the expression of ABI3 exhibit negatively correlated with the expression of MMR genes, suggesting that ABI3 may reduce TMB and MSI through the MMR system in certain cancers, such as BRCA. Therefore, in the future, we can estimate the effect of immunotherapy by detecting the expression level of ABI3, and develop personalized therapy for ABI3 to combine with conventional immunotherapy to enhance its efficacy.

Our investigation also revealed that ABI3 has a pivotal function in oncological immunity. The characteristics of TME have the potential to serve as indicators for evaluating the reaction of tumor cells to immunological treatment, and have an impact on clinical outcomes (Murciano-Goroff et al., 2020). Typically, the immune system has the capacity to detect and eradicate tumor cells within the TME. Nevertheless, tumor cells may utilize various survival and proliferation mechanisms, eluding the immune system. According to ESTIMATE, ABI3 expression manifested a positive correlation with stromal scores, immune scores, and ESTIMATE scores in the TME of 33 cancers, indicating that ABI3 was an important player in the immune response to cancer.

Numerous studies have revealed that the tumor microenvironment exerts a profound influence not only on tumor growth but also on immune escape and treatment resistance. Immune cells infiltrating tumors, a crucial constituent of the tumor microenvironment, can either counteract or facilitate tumor development and progression (Lei et al., 2020). Consequently, we investigated the relationship between ABI3 expression and immune cell infiltration. One of the significant outcomes of our study was the robust correlation between ABI3 expression and immune infiltration among various cancer types. Notably, ABI3 expression exhibited a positive correlation with the degree of infiltration by CD4^+^ T cells, CAF, Endo, HSC, γ/δT, NK T cells, Tregs, B cells, Monocytes, and CD8^+^ T cells in the majority of cancers, suggesting that ABI3 potentially influences cancer progression and outcome by modulating the tumor microenvironment. In accordance with a study by Wang et al. (2017b), elevated M2 macrophage infiltration has been connected to short-term relapse and a poorer prognosis in LGG in multiple clinical trials, which is consistent with our prognostic analysis. Thus, we postulate that ABI3 may operate in LGG patients by adjusting the quantity of M2 macrophages in the tumor microenvironment, which further contributes to a worse prognosis.

Moreover, our investigation offers supplementary perspectives on the wider applicability of ABI3 in tumors and verifies that ABI3 expression was intimately connected to immune cells and immune-related molecules across the majority of cancers. Our correlation analysis of ABI3 and immunoreactive genes across cancers demonstrated a positive relationship between ABI3 expression and genes related to immunoregulation, including MHC, immune activation, immunosuppressive, chemokine, and chemokine receptor proteins, in nearly all cancer types. Collectively, these findings imply that ABI3 expression was tightly interwoven with immune infiltration of tumor cells, affects patient prognosis, and introduces novel targets for the creation of immunomodulatory agents.

The cBioPortal database reveals that the frequency of ABI3 alterations was highest among pan-cancer patients with BRCA. Regarding mutation-related survival analysis, ABI3 gene mutations in LGG patients display the most significant changes in all survival indicators. According to the HPA database, high expression of ABI3 in UCEC patients was favorable for patient prognosis and was considered a favorable factor. Hence, these three types of cancers were selected for subsequent enrichment analysis. GSEA enrichment analysis revealed that high ABI3 expression was predominantly associated with immune-activated processes, such as Allograft rejection, Autoimmune thyroid disease, and Graft−versus−host disease. Since most cancers rely on reactivation of telomerase, combined with the analysis of ABI3 survival results in BRCA, high ABI3 expression may contribute to the inhibition of telomerase-related activities, indicating that ABI3 may play an indispensable role in oncogenesis and cancer development by participating in the regulation of telomerase activity (O'Sullivan and Telomeres, 2010). DNA replication and cancer progression are closely linked, and the inability of cells to cope with replication stress has been reported to have oncogenic effects (Gaillard et al., 2015). Consequently, ABI3 may influence the disease progression of UCEC patients by impacting the DNA replication process. While the literature has examined the relationship between neural activity, synaptic function, and cancer, few studies have directly addressed the relationship between synaptic vesicle activity and cancer development (Venkatesh and Monje, 2017). A prior investigation has demonstrated that members of ABI are crucial for synapse formation (Proepper et al., 2007), suggesting that the specific relationship between ABI3 and synaptic vesicle-related activity in LGG cancer enrichment results warrants further exploration. Ultimately, these findings suggest that increased ABI3 expression was associated with the immune-activated condition of malignancies and may be involved in the regulation of telomerase or DNA-related functions, ultimately influencing processes such as cancer initiation and progression.

Subsequently, we conducted a screening for potential anti-cancer drugs that may exert their therapeutic effects through the action of ABI3. This approach could potentially guide drug selection based on the expression of ABI3 in cancer. Sensitivity analysis of potential drugs targeting ABI3 revealed that Megestrol acetate (MA) holds great potential for developing new molecular targets. Megestrol acetate is a semi-synthetic progestin that has demonstrated potential for treating endometrial cancer and prostate cancer (Schacter et al., 1989) and has long been used for the treatment of breast cancer due to its dose-dependent effect (Sedlacek, 1988; Bines et al., 2014). Its anti-tumor activity may involve interaction with steroid receptors, leading to physiological effects akin to natural progesterone (Lonning, 2009). With these aspects in mind, we assumed that the therapeutic effects of Megestrol acetate may be attributed to its targeting of ABI3. Furthermore, our findings have unveiled ABI3 as a promising novel target for the development of anti-BRCA drugs.

Admittedly, despite our exploration and incorporation of information from multiple databases, the present study still encountered limitations. Our research analyzed transcriptomic data collected from openly accessible databases, which unavoidably introduces methodological bias; further *in vitro* or *in vivo* biological experiments are necessary to validate our findings and enhance clinical application.

In conclusion, through multi-omics analyses of ABI3 across various cancer types, we identified it as a promising prognostic indicator. ABI3 expression exhibited varying correlations with immune regulatory factors, immune cell infiltration, tumor microenvironment, TMB, and MSI across different cancer types, influencing tumor immunity differently. This investigation highlights the crucial role of ABI3 in tumorigenesis and metastasis, as well as its impact on the immunological and metabolic activity of malignant tumors. To enable more precise and personalized immunotherapy for specific cancers, such as BRCA, future research should focus on clarifying the expression of ABI3 in cancer patients.

## Data Availability

The original contributions presented in the study are included in the article/Supplementary Material, further inquiries can be directed to the corresponding authors.

## References

[B1] Bai K. H. Zhang Y. Y. Li X. P. Tian X. P. Pan M. M. Wang D. W. (2022). Comprehensive analysis of tumor necrosis factor-α-inducible protein 8-like 2 (TIPE2): a potential novel pan-cancer immune checkpoint. Comput. Struct. Biotechnol. J. 20, 5226–5234. Epub 20220917. 10.1016/j.csbj.2022.09.021 36187930 PMC9508481

[B2] Baretti M. Le D. T. (2018). DNA mismatch repair in cancer. Pharmacol. Ther. 189, 45–62. Epub 20180415. 10.1016/j.pharmthera.2018.04.004 29669262

[B3] Bines J. Dienstmann R. Obadia R. M. Branco L. G. P. Quintella D. C. Castro T. M. (2014). Activity of Megestrol acetate in postmenopausal women with advanced breast cancer after nonsteroidal aromatase inhibitor failure: a phase ii trial. Ann. Oncol. 25 (4), 831–836. Epub 20140310. 10.1093/annonc/mdu015 24615412

[B4] Blum A. Wang P. Zenklusen J. C. (2018). Snapshot: tcga-analyzed tumors. Cell 173 (2), 530. 10.1016/j.cell.2018.03.059 29625059

[B5] Bonneville R. Krook M. A. Kautto E. A. Miya J. Wing M. R. Chen H. Z. (2017). Landscape of microsatellite instability across 39 cancer types. JCO Precis Oncol. 20171003. 10.1200/PO.17.00073 PMC5972025 29850653

[B6] Borst J. Ahrends T. Babala N. Melief C. J. M. Kastenmuller W. (2018). Cd4(+) T cell help in cancer immunology and immunotherapy. Nat. Rev. Immunol. 18 (10), 635–647. 10.1038/s41577-018-0044-0 30057419

[B7] Chen B. Brinkmann K. Chen Z. Pak C. W. Liao Y. Shi S. (2014). The wave regulatory complex links diverse receptors to the actin cytoskeleton. Cell 156 (1-2), 195–207. 10.1016/j.cell.2013.11.048 24439376 PMC4059610

[B8] Chorzalska A. Salloum I. Shafqat H. Khan S. Marjon P. Treaba D. (2014). Low expression of Abelson interactor-1 is linked to acquired drug resistance in bcr-abl-induced leukemia. Leukemia 28 (11), 2165–2177. Epub 20140404. 10.1038/leu.2014.120 24699303 PMC4185277

[B9] Choucair K. Morand S. Stanbery L. Edelman G. Dworkin L. Nemunaitis J. (2020). Tmb: a promising immune-response biomarker, and potential spearhead in advancing targeted therapy trials. Cancer Gene Ther. 27 (12), 841–853. Epub 20200428. 10.1038/s41417-020-0174-y 32341410

[B10] Cui M. Yu W. Dong J. Chen J. Zhang X. Liu Y. (2010). Downregulation of Abi1 expression affects the progression and prognosis of human gastric carcinoma. Med. Oncol. 27 (3), 632–639. Epub 20090625. 10.1007/s12032-009-9260-6 19554484

[B11] Dudley J. C. Lin M. T. Le D. T. Eshleman J. R. (2016). Microsatellite instability as a biomarker for Pd-1 blockade. Clin. Cancer Res. 22 (4), 813–820. 10.1158/1078-0432.CCR-15-1678 26880610

[B12] Eden S. Rohatgi R. Podtelejnikov A. V. Mann M. Kirschner M. W. (2002). Mechanism of regulation of wave1-induced actin nucleation by Rac1 and Nck. Nature 418 (6899), 790–793. 10.1038/nature00859 12181570

[B13] Evan G. I. Vousden K. H. (2001). Proliferation, cell cycle and apoptosis in cancer. Nature 411 (6835), 342–348. 10.1038/35077213 11357141

[B14] Feng Y. Tao F. Qiao H. Tang H. (2023). A pan-cancer analysis of Abi3bp: a potential biomarker for prognosis and immunoinfiltration. Front. Oncol. 13, 1159725. Epub 20230501. 10.3389/fonc.2023.1159725 37197424 PMC10183607

[B15] Ferlay J. Colombet M. Soerjomataram I. Parkin D. M. Pineros M. Znaor A. (2021). Cancer statistics for the year 2020: an overview. Int J Cancer. 20210405. 10.1002/ijc.33588 33818764

[B16] Gaillard H. Garcia-Muse T. Aguilera A. (2015). Replication stress and cancer. Nat. Rev. Cancer 15 (5), 276–289. 10.1038/nrc3916 25907220

[B17] Galbo P. M. Jr. Zang X. Zheng D. (2021). Molecular features of cancer-associated fibroblast subtypes and their implication on cancer pathogenesis, prognosis, and immunotherapy resistance. Clin. Cancer Res. 27 (9), 2636–2647. Epub 20210223. 10.1158/1078-0432.CCR-20-4226 33622705 PMC8102353

[B18] Grove M. Demyanenko G. Echarri A. Zipfel P. A. Quiroz M. E. Rodriguiz R. M. (2004). Abi2-Deficient mice exhibit defective cell migration, aberrant dendritic spine morphogenesis, and deficits in learning and memory. Mol. Cell Biol. 24 (24), 10905–10922. 10.1128/MCB.24.24.10905-10922.2004 15572692 PMC533973

[B19] Hinshaw D. C. Shevde L. A. (2019). The tumor microenvironment innately modulates cancer progression. Cancer Res. 79 (18), 4557–4566. Epub 20190726. 10.1158/0008-5472.CAN-18-3962 31350295 PMC6744958

[B20] Ichigotani Y. Yokozaki S. Fukuda Y. Hamaguchi M. Matsuda S. (2002). Forced expression of nesh suppresses motility and metastatic dissemination of malignant cells. Cancer Res. 62 (8), 2215–2219. 11956071

[B21] Innocenti M. Zucconi A. Disanza A. Frittoli E. Areces L. B. Steffen A. (2004). Abi1 is essential for the formation and activation of a Wave2 signalling complex. Nat. Cell Biol. 6 (4), 319–327. Epub 20040328. 10.1038/ncb1105 15048123

[B22] Jumper J. Evans R. Pritzel A. Green T. Figurnov M. Ronneberger O. (2021). Highly accurate protein structure prediction with alphafold. Nature 596 (7873), 583–589. Epub 20210715. 10.1038/s41586-021-03819-2 34265844 PMC8371605

[B23] Kanduri M. Cahill N. Goransson H. Enstrom C. Ryan F. Isaksson A. (2010). Differential genome-wide array-based methylation profiles in prognostic subsets of chronic lymphocytic leukemia. Blood 115 (2), 296–305. Epub 20091106. 10.1182/blood-2009-07-232868 19897574

[B24] Kim S. Chen J. Cheng T. Gindulyte A. He J. He S. (2021). Pubchem in 2021: new data content and improved web interfaces. Nucleic Acids Res. 49 (D1), D1388–D1395. 10.1093/nar/gkaa971 33151290 PMC7778930

[B25] Kulis M. Esteller M. (2010). DNA methylation and cancer. Adv. Genet. 70, 27–56. 10.1016/B978-0-12-380866-0.60002-2 20920744

[B26] Kurisu S. Suetsugu S. Yamazaki D. Yamaguchi H. Takenawa T. (2005). Rac-Wave2 signaling is involved in the invasive and metastatic phenotypes of murine melanoma cells. Oncogene 24 (8), 1309–1319. 10.1038/sj.onc.1208177 15608687

[B27] Li T. Fu J. Zeng Z. Cohen D. Li J. Chen Q. (2020). Timer2.0 for analysis of tumor-infiltrating immune cells. Nucleic Acids Res. 48 (W1), W509-W514. 10.1093/nar/gkaa407 32442275 PMC7319575

[B28] Latini F. R. Hemerly J. P. Freitas B. C. Oler G. Riggins G. J. Cerutti J. M. (2011). Abi3 ectopic expression reduces *in vitro* and *in vivo* cell growth properties while inducing senescence. BMC Cancer 11, 11. Epub 20110111. 10.1186/1471-2407-11-11 21223585 PMC3032749

[B29] Law A. L. Vehlow A. Kotini M. Dodgson L. Soong D. Theveneau E. (2013). Lamellipodin and the scar/wave complex cooperate to promote cell migration *in vivo* . J. Cell Biol. 203 (4), 673–689. Epub 20131118. 10.1083/jcb.201304051 24247431 PMC3840943

[B30] Lei X. Lei Y. Li J. K. Du W. X. Li R. G. Yang J. (2020). Immune cells within the tumor microenvironment: biological functions and roles in cancer immunotherapy. Cancer Lett. 470, 126–133. Epub 20191112. 10.1016/j.canlet.2019.11.009 31730903

[B31] Leng Y. Zhang J. Badour K. Arpaia E. Freeman S. Cheung P. (2005). Abelson-Interactor-1 promotes Wave2 membrane translocation and abelson-mediated tyrosine phosphorylation required for Wave2 activation. Proc. Natl. Acad. Sci. U. S. A. 102 (4), 1098–1103. Epub 20050118. 10.1073/pnas.0409120102 15657136 PMC545868

[B32] Lin T. C. (2022). Runx1 and cancer. Biochim. Biophys. Acta Rev. Cancer 1877 (3), 188715. Epub 20220307. 10.1016/j.bbcan.2022.188715 35271994

[B33] Liu C. J. Hu F. F. Xia M. X. Han L. Zhang Q. Guo A. Y. (2018). Gscalite: a web server for gene set cancer analysis. Bioinformatics 34 (21), 3771–3772. 10.1093/bioinformatics/bty411 29790900

[B34] Lonning P. E. (2009). Additive endocrine therapy for advanced breast cancer - back to the future. Acta Oncol. 48 (8), 1092–1101. 10.3109/02841860903117816 19863216

[B35] Matsuda S. Yokozaki S. Yoshida H. Kitagishi Y. Shirafuji N. Okumura N. (2008). Insulin receptor substrate protein 53 (Irsp53) as a binding partner of antimetastasis molecule nesh, a member of Abelson interactor protein family. Ann. Oncol. 19 (7), 1356–1357. Epub 20080513. 10.1093/annonc/mdn293 18480067

[B36] Miyazaki K. Matsuda S. Ichigotani Y. Takenouchi Y. Hayashi K. Fukuda Y. (2000). Isolation and characterization of a novel human gene (nesh) which encodes a putative signaling molecule similar to E3b1 protein. Biochim. Biophys. Acta 1493 (1-2), 237–241. 10.1016/s0167-4781(00)00158-5 10978530

[B37] Morris G. M. Huey R. Lindstrom W. Sanner M. F. Belew R. K. Goodsell D. S. (2009). Autodock4 and Autodocktools4: automated docking with selective receptor flexibility. J. Comput. Chem. 30 (16), 2785–2791. 10.1002/jcc.21256 19399780 PMC2760638

[B38] Murciano-Goroff Y. R. Warner A. B. Wolchok J. D. (2020). The future of cancer immunotherapy: microenvironment-targeting combinations. Cell Res. 30 (6), 507–519. Epub 20200528. 10.1038/s41422-020-0337-2 32467593 PMC7264181

[B39] Nusinow D. P. Szpyt J. Ghandi M. Rose C. M. McDonald E. R. 3rd Kalocsay M. (2020). Quantitative proteomics of the cancer cell line Encyclopedia. Cell 180 (2), 387–402. 10.1016/j.cell.2019.12.023 31978347 PMC7339254

[B40] O'Sullivan R. J. Telomeres K. J. (2010). Telomeres: protecting chromosomes against genome instability. Nat. Rev. Mol. Cell Biol. 11 (3), 171–181. Epub 20100203. 10.1038/nrm2848 20125188 PMC2842081

[B41] Pathania R. Ramachandran S. Elangovan S. Padia R. Yang P. Cinghu S. (2015). Dnmt1 is essential for mammary and cancer stem cell maintenance and tumorigenesis. Nat. Commun. 6, 6910. Epub 20150424. 10.1038/ncomms7910 25908435 PMC4410389

[B42] Proepper C. Johannsen S. Liebau S. Dahl J. Vaida B. Bockmann J. (2007). Abelson interacting protein 1 (Abi-1) is essential for dendrite morphogenesis and synapse formation. EMBO J. 26 (5), 1397–1409. Epub 20070215. 10.1038/sj.emboj.7601569 17304222 PMC1817621

[B43] Reinhold W. C. Sunshine M. Liu H. Varma S. Kohn K. W. Morris J. (2012). Cellminer: a web-based suite of genomic and pharmacologic tools to explore transcript and drug patterns in the nci-60 cell line set. Cancer Res. 72 (14), 3499–3511. 10.1158/0008-5472.CAN-12-1370 22802077 PMC3399763

[B44] Ru B. Wong C. N. Tong Y. Zhong J. Y. Zhong S. S. W. Wu W. C. (2019). Tisidb: an integrated repository portal for tumor-immune system interactions. Bioinformatics 35 (20), 4200–4202. 10.1093/bioinformatics/btz210 30903160

[B45] Samstein R. M. Lee C. H. Shoushtari A. N. Hellmann M. D. Shen R. Janjigian Y. Y. (2019). Tumor mutational load predicts survival after immunotherapy across multiple cancer types. Nat. Genet. 51 (2), 202–206. Epub 20190114. 10.1038/s41588-018-0312-8 30643254 PMC6365097

[B46] Schacter L. Rozencweig M. Canetta R. Kelley S. Nicaise C. Smaldone L. (1989). Megestrol acetate: clinical experience. Cancer Treat. Rev. 16 (1), 49–63. 10.1016/0305-7372(89)90004-2 2471590

[B47] Schaks M. Giannone G. Rottner K. (2019). Actin dynamics in cell migration. Essays Biochem. 63 (5), 483–495. 10.1042/EBC20190015 31551324 PMC6823167

[B48] Sedlacek S. M. (1988). An overview of Megestrol acetate for the treatment of advanced breast cancer. Semin. Oncol. 15 (2), 3–13. 3285483

[B49] Siegel R. L. Miller K. D. Wagle N. S. Jemal A. (2023). Cancer statistics, 2023. CA Cancer J. Clin. 73 (1), 17–48. 10.3322/caac.21763 36633525

[B50] Steinestel K. Bruderlein S. Lennerz J. K. Steinestel J. Kraft K. Propper C. (2014). Expression and Y435-phosphorylation of Abelson interactor 1 (Abi1) promotes tumour cell adhesion, extracellular matrix degradation and invasion by colorectal carcinoma cells. Mol. Cancer 13, 145. Epub 20140609. 10.1186/1476-4598-13-145 24913355 PMC4066275

[B51] Steuer C. E. Ramalingam S. S. (2018). Tumor mutation burden: leading immunotherapy to the era of precision medicine? J. Clin. Oncol. 36 (7), 631–632. Epub 20180116. 10.1200/JCO.2017.76.8770 29337637

[B52] Stierand K. Maass P. C. Rarey M. (2006). Molecular complexes at a glance: automated generation of two-dimensional complex diagrams. Bioinformatics 22 (14), 1710–1716. Epub 20060421. 10.1093/bioinformatics/btl150 16632493

[B53] Sun D. Wang J. Han Y. Dong X. Ge J. Zheng R. (2021). Tisch: a comprehensive web Resource enabling interactive single-cell transcriptome visualization of tumor microenvironment. Nucleic Acids Res. 49 (D1), D1420–D1430. 10.1093/nar/gkaa1020 33179754 PMC7778907

[B54] Thorsson V. Gibbs D. L. Brown S. D. Wolf D. Bortone D. S. Ou Yang T. H. (2018). The immune landscape of cancer. Immunity 48 (4), 812–830.e14. Epub 20180405. 10.1016/j.immuni.2018.03.023 29628290 PMC5982584

[B55] Tomczak K. Czerwinska P. Wiznerowicz M. (2015). The cancer genome Atlas (tcga): an immeasurable source of knowledge. Contemp. Oncol. Pozn. 19 (1A), A68–A77. 10.5114/wo.2014.47136 25691825 PMC4322527

[B56] Tsai J. H. Yang J. (2013). Epithelial-mesenchymal plasticity in carcinoma metastasis. Genes Dev. 27 (20), 2192–2206. 10.1101/gad.225334.113 24142872 PMC3814640

[B57] Tuck M. Janssens M. (1990). The differential efficacy of antihypertensive agents in the elderly. J. Hum. Hypertens. 4 (4), 415–420. 2258887

[B58] van Velzen M. J. M. Derks S. van Grieken N. C. T. Haj Mohammad N. van Laarhoven H. W. M. (2020). Msi as a predictive factor for treatment outcome of gastroesophageal adenocarcinoma, Cancer Treat Rev 86. 102024. Epub 20200428. 10.1016/j.ctrv.2020.102024 32388292

[B59] Venkatesh H. Monje M. (2017). Neuronal activity in ontogeny and oncology. Trends Cancer 3 (2), 89–112. Epub 20170213. 10.1016/j.trecan.2016.12.008 28718448 PMC5518622

[B60] Vivian J. Rao A. A. Nothaft F. A. Ketchum C. Armstrong J. Novak A. (2017). Toil enables reproducible, open source, big biomedical data analyses. Nat. Biotechnol. 35 (4), 314–316. 10.1038/nbt.3772 28398314 PMC5546205

[B61] Wang C. Navab R. Iakovlev V. Leng Y. Zhang J. Tsao M. S. (2007). Abelson interactor protein-1 positively regulates breast cancer cell proliferation, migration, and invasion. Mol. Cancer Res. 5 (10), 1031–1039. 10.1158/1541-7786.MCR-06-0391 17951403

[B62] Wang H. Kaur G. Sankin A. I. Chen F. Guan F. Zang X. (2019). Immune checkpoint blockade and car-T cell therapy in hematologic malignancies. J. Hematol. Oncol. 12 (1), 59. Epub 20190611. 10.1186/s13045-019-0746-1 31186046 PMC6558778

[B63] Wang J. L. Yan T. T. Long C. Cai W. W. (2017a). Oncogenic function and prognostic significance of Abelson interactor 1 in hepatocellular carcinoma. Int. J. Oncol. 50 (5), 1889–1898. Epub 20170320. 10.3892/ijo.2017.3920 28339046

[B64] Wang Q. Hu B. Hu X. Kim H. Squatrito M. Scarpace L. (2017b). Tumor evolution of glioma-intrinsic gene expression subtypes associates with immunological changes in the microenvironment. Cancer Cell 32 (1), 42–56. 10.1016/j.ccell.2017.06.003 28697342 PMC5599156

[B65] Warde-Farley D. Donaldson S. L. Comes O. Zuberi K. Badrawi R. Chao P. (2010). The genemania prediction server: biological network integration for gene prioritization and predicting gene function. Nucleic Acids Res. 38, W214–W220. Web Server issue). 10.1093/nar/gkq537 20576703 PMC2896186

[B66] Wu T. Hu E. Xu S. Chen M. Guo P. Dai Z. (2021). Clusterprofiler 4.0: a universal enrichment tool for interpreting omics data. Innov. (Camb) 2 (3), 100141. Epub 20210701. 10.1016/j.xinn.2021.100141 PMC8454663 34557778

[B67] Yang X. Ding Y. Sun L. Shi M. Zhang P. He A. (2022). Wasf2 serves as a potential biomarker and therapeutic target in ovarian cancer: a pan-cancer analysis. Front. Oncol. 12, 840038. Epub 20220314. 10.3389/fonc.2022.840038 35359421 PMC8964075

[B68] Yoshihara K. Shahmoradgoli M. Martinez E. Vegesna R. Kim H. Torres-Garcia W. (2013). Inferring tumour purity and stromal and immune cell admixture from expression data. Nat. Commun. 4, 2612. 10.1038/ncomms3612 24113773 PMC3826632

[B69] Yu W. Sun X. Clough N. Cobos E. Tao Y. Dai Z. (2008). Abi1 gene silencing by short hairpin rna impairs bcr-abl-induced cell adhesion and migration *in vitro* and leukemogenesis *in vivo* . Carcinogenesis 29 (9), 1717–1724. Epub 20080502. 10.1093/carcin/bgn098 18453543 PMC2527646

[B70] Zhang Y. Zhong Z. Li M. Chen J. Lin T. Sun J. (2021). The roles and prognostic significance of abi1-tsv-11 expression in patients with left-sided colorectal cancer. Sci. Rep. 11 (1), 10734. Epub 20210524. 10.1038/s41598-021-90220-8 34031495 PMC8144562

